# A Feature-Enhanced Multi-Kernel Adaptation Framework for Cross-Domain Fault Diagnosis of Ball Screw Feed Systems

**DOI:** 10.3390/s26196291

**Published:** 2026-10-04

**Authors:** Xiao-Yi Wang, Jing-Lun Xie, Chang-Guang Zhou, Hua-Xi Zhou, Chen Yin

**Affiliations:** 1 Department of Mechanical Engineering, Wuhan Polytechnic University, Wuhan 430048, China; 2 Department of Mechanical Engineering, Nanjing University of Science and Technology, Nanjing 210094, China; 3 Department of Data Science, College of Computing, City University of Hong Kong, Kowloon, Hong Kong; 4 City University of Hong Kong Shenzhen Research Institute, Shenzhen 518000, China

**Keywords:** ball screw feed system, local spalling fault, feature enhancement, cross-domain fault identification, domain adaptation, multi-kernel maximum mean discrepancy

## Abstract

Ball screw feed systems are critical components of CNC machine tools, and their health condition directly affects positioning accuracy and operational reliability. However, variations in rotational speed under changing operating conditions can cause substantial shifts in fault characteristics, limiting the generalization capability of conventional fault identification methods developed under a single operating condition. To address this issue, a feature-enhanced multi-kernel domain adaptation framework is proposed for cross-domain fault identification of ball screw feed systems. First, an attention-based feature enhancement module is developed to adaptively reweight vibration features, thereby emphasizing informative feature components and suppressing redundant and noise-related information. Subsequently, a residual feature extraction and fusion module is employed to learn hierarchical fault representations and improve feature discriminability. Finally, a multi-kernel maximum mean discrepancy (MK-MMD) strategy is integrated with adversarial domain adaptation to reduce feature distribution discrepancies between source and target domains under different rotational speeds and ball screw specimens. Local spalling fault experiments were conducted on the developed test bench, where vibration signals were collected using an accelerometer under various operating conditions and subsequently used to validate the proposed framework. Experimental results demonstrate that the proposed method achieves an average identification accuracy of 92.9% across the 12 investigated cross-domain tasks, compared with 69.7% for the optimized source-only CNN, with the performance gap becoming particularly pronounced under cross-specimen transfer conditions. The results indicate that the proposed framework improves the learning of fault-discriminative and domain-invariant representations, thereby improving the robustness of ball screw fault identification under variable operating conditions.

## 1. Introduction

Ball screw feed systems are critical transmission and actuation components in computer numerical control (CNC) machine tools, industrial robots, and electromechanical servo systems, where they convert rotary motion into high-precision linear motion. Their positioning accuracy, stiffness, and operational reliability directly influence machining quality and the overall performance of manufacturing equipment. Previous studies have indicated that a considerable proportion of machine tool failures are associated with degradation of feed drive systems, while ball screw mechanisms represent one of the vulnerable components in electromechanical transmission systems [1,2,3]. In aerospace, automotive, and other high-end manufacturing industries, unexpected ball screw failures may lead to production interruptions, reduced machining accuracy, and even safety risks [4,5]. Therefore, reliable fault identification and condition monitoring of ball screw feed systems are of great significance for ensuring equipment reliability and enabling predictive maintenance.

Existing fault diagnosis approaches for ball screw systems can generally be classified into physics-based and data-driven methods. Physics-based approaches establish dynamic models incorporating contact deformation, load distribution, and ball circulation characteristics to reveal the relationship between fault mechanisms and system responses [6,7,8,9]. However, physics-based models generally rely on idealized assumptions regarding boundary conditions, material properties, and contact states, making it difficult to fully describe the nonlinear, time-varying, and multi-factor coupled behaviors encountered under practical operating conditions. In contrast, data-driven methods directly utilize measured sensor signals and perform fault identification through signal processing and pattern recognition techniques. Among various sensing approaches, vibration analysis has become one of the most widely adopted methods due to its high sensitivity to mechanical abnormalities and rich fault-related information [10]. Guo et al. [11] combined outlier detection with instantaneous frequency estimation to achieve fault localization under variable-speed conditions. Zhou et al. [12] employed ensemble empirical mode decomposition, linear discriminant analysis, and support vector machines to classify degradation states under different preload conditions. Tsai et al. [13] monitored variations in ball-passing frequency to identify the occurrence of preload loss. Nguyen et al. [14] established an online preload monitoring method by analyzing the frequency response characteristics of motor current and nut vibration signals. Kundu et al. [15] introduced variational mode decomposition into ball screw anomaly detection and achieved early identification of preload degradation. Nevertheless, these approaches rely heavily on manually designed features and shallow classifiers, which limits their capability to characterize complex nonlinear fault representations. More importantly, the learned features are often sensitive to changes in operating conditions and measurement noise, resulting in degraded diagnostic performance when the trained model is applied to unseen domains.

With the rapid development of artificial intelligence, deep learning methods, including convolutional neural networks (CNNs), long short-term memory networks (LSTMs), and autoencoders, have been increasingly applied to ball screw fault diagnosis because of their powerful automatic feature extraction capabilities [16,17]. Lei et al. [18] proposed a CNN-LSTM model to extract temporal features from full-life-cycle vibration signals for performance degradation estimation. Yin et al. [19] combined one-dimensional CNN with orthogonal experimental design to improve the diagnosis accuracy of early lubrication degradation. Shan et al. [20] further enhanced multi-fault identification through CNN-based multisensor feature fusion. Benker and Zaeh [21] demonstrated that CNN models could achieve high recognition accuracy on unseen components, while Zhang et al. [22] achieved accurate diagnosis through multimodal signal fusion. These studies demonstrate the significant potential of deep learning for automatic fault identification. However, two major challenges remain. First, vibration responses of ball screw feed systems exhibit strong dependence on operating conditions. Even under identical fault states, variations in rotational speed and specimen characteristics can cause shifts in feature amplitudes, frequency components, and feature distributions, resulting in significant domain discrepancies. Second, fault data collected under practical operating conditions are often limited and expensive to annotate, making it difficult to obtain sufficient labeled samples for every possible operating condition.

To overcome the limitations associated with domain shifts and insufficient labeled data, transfer learning and domain adaptation techniques have recently been introduced into mechanical fault diagnosis. The fundamental idea is to transfer diagnostic knowledge learned from labeled source-domain data to an unlabeled or weakly labeled target domain, thereby improving fault identification performance under different operating conditions [23]. Zhang et al. [24] proposed an instance-based ensemble deep transfer learning network for multi-condition degradation recognition. Azamfar et al. [25] developed a CNN-based domain adaptation approach using maximum mean discrepancy (MMD) for cross-condition fault diagnosis of ball screw systems. Pandhare et al. [26] proposed a CNN-based domain adaptation method using MMD for cross-sensor ball screw fault diagnosis. Zhou et al. [27] developed an SE-Resformer-based transfer learning framework with domain adversarial training, which maintained reliable diagnosis performance under strong noise interference. Li et al. [28] proposed a lightweight diagnostic network with joint distribution alignment for heterogeneous sensing conditions. These studies demonstrate that domain adaptation provides an effective solution for fault identification under variable operating conditions. However, existing approaches mainly focus on reducing domain discrepancies, while the discriminability of the input vibration representations may still be limited by noise and redundant feature components. In addition, the use of a single kernel for distribution measurement may not adequately characterize complex feature discrepancies across different operating conditions and ball screw specimens. Therefore, an effective framework that simultaneously improves feature discriminability and provides robust multi-kernel distribution alignment is still required for cross-domain fault identification of ball screw feed systems.

Although several domain adaptation methods have been developed for cross-domain ball screw fault diagnosis, their feature representation and domain-alignment strategies differ considerably. To better clarify the incremental contribution of the present study, representative closely related methods are compared in Table 1. Compared with existing MMD-based domain adaptation methods, the proposed FE-MK-DANN further integrates attention-based feature enhancement, residual feature extraction, adversarial alignment, and MK-MMD, and is evaluated under both cross-speed and cross-specimen transfer scenarios.

In light of the above challenges, this study proposes a feature-enhanced multi-kernel domain adversarial neural network (FE-MK-DANN) for cross-domain fault identification of ball screw feed systems. First, an attention-based feature enhancement module is developed to adaptively reweight vibration feature components, thereby enhancing informative representations and suppressing redundant and noise-related information. Second, a residual feature extraction and fusion module is constructed to learn hierarchical fault representations and improve feature propagation and discriminability. Third, a multi-kernel maximum mean discrepancy (MK-MMD) strategy is incorporated into an adversarial domain adaptation framework to characterize and reduce distribution discrepancies between source and target domains from multiple reproducing kernel Hilbert spaces (RKHSs). Through the joint optimization of fault classification and domain adaptation objectives, the proposed framework aims to learn representations that are simultaneously fault-discriminative and domain-invariant. The effectiveness of the proposed method is experimentally validated using local spalling faults under multiple rotational speeds and ball screw specimens. It should be noted that the cross-domain fault identification problem considered in this study follows a transductive unsupervised domain adaptation setting. The framework assumes that labeled source-domain data and unlabeled target-domain samples are available during adaptation, whereas no target-domain fault labels are used. This differs from domain generalization, where target-domain data are unavailable during model development, and from inductive deployment scenarios in which the trained model is directly applied to unseen target samples without target-specific adaptation. In practical ball-screw maintenance, unlabeled vibration measurements, particularly those collected during normal operation, are generally much easier to obtain than labeled fault samples, providing a practical basis for such target-domain adaptation.

## 2. Preliminaries

### 2.1. Fundamentals of CNN

**Figure 1:**
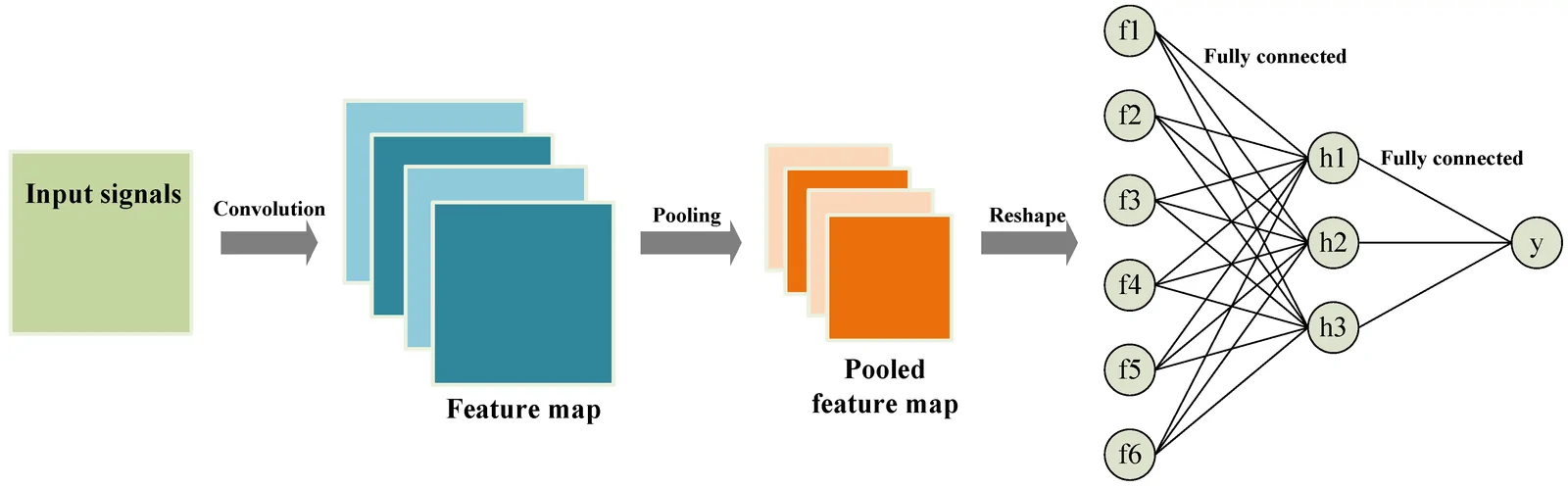
Convolutional Neural Network architecture.

A convolutional neural network (CNN) is a representative deep learning architecture consisting of multiple hierarchical layers for automatic feature extraction, feature representation, and pattern recognition. Owing to its powerful capability in capturing spatial and local correlations, CNNs have been widely applied in various fields, including image processing, speech recognition, and mechanical signal analysis. A typical CNN architecture mainly comprises convolutional layers, activation functions, pooling layers, and fully connected layers, as illustrated in Figure 1. During the feature learning process, the input signals are first transformed into feature maps through convolution operations, where learnable kernels capture local characteristics and spatial dependencies. Subsequently, activation functions introduce nonlinear transformations to enhance the representation capability of the network. Pooling operations are then employed to reduce feature dimensionality and improve computational efficiency while retaining essential information. Finally, the fully connected layers integrate the extracted high-level features and perform mapping to the target outputs for classification or regression tasks.

The convolutional layer serves as the fundamental component of CNNs and is responsible for extracting local feature representations from the input data through learnable convolution kernels. By sliding kernels over the input signals, convolution operations capture local patterns and generate feature maps, which can be expressed as shown in Equation (1).

(1) $$Y(i,j)=\sum_{m,n}X(i-m,j-n)\cdot W(m,n)+b$$

where *Y* denotes the generated feature map, *X* represents the input feature representation, *W* denotes the learnable weights of the convolution kernel, and *b* denotes the bias term.

The pooling layer is introduced to reduce feature dimensionality and computational complexity while preserving the most representative information. Common pooling strategies include max pooling and average pooling. Specifically, max pooling selects the maximum response within a predefined local region, whereas average pooling calculates the mean value of the corresponding region.

Activation functions play a critical role in introducing nonlinearity into CNN architectures, enabling the networks to approximate complex nonlinear relationships. Commonly used activation functions include the rectified linear unit (ReLU), hyperbolic tangent (Tanh), sigmoid, and Mish functions. Among them, the ReLU function is defined as

(2) $$Y(i,j)=\left\{\begin{array}{l} X(i,j)\;\;\;\;X(i,j)\geq 0\\ 0\;\;\;\;\;\;\;\;X(i,j)<0 \end{array}\right.$$

The fully connected layer is generally employed for high-level feature integration and decision-making. After hierarchical feature extraction through convolutional and pooling operations, the fully connected layer performs weighted aggregation of the extracted features and maps them into the corresponding output space for classification or regression tasks.

### 2.2. Attention Mechanism and Residual Network Architecture

#### 2.2.1. Attention Mechanism

**Figure 2:**
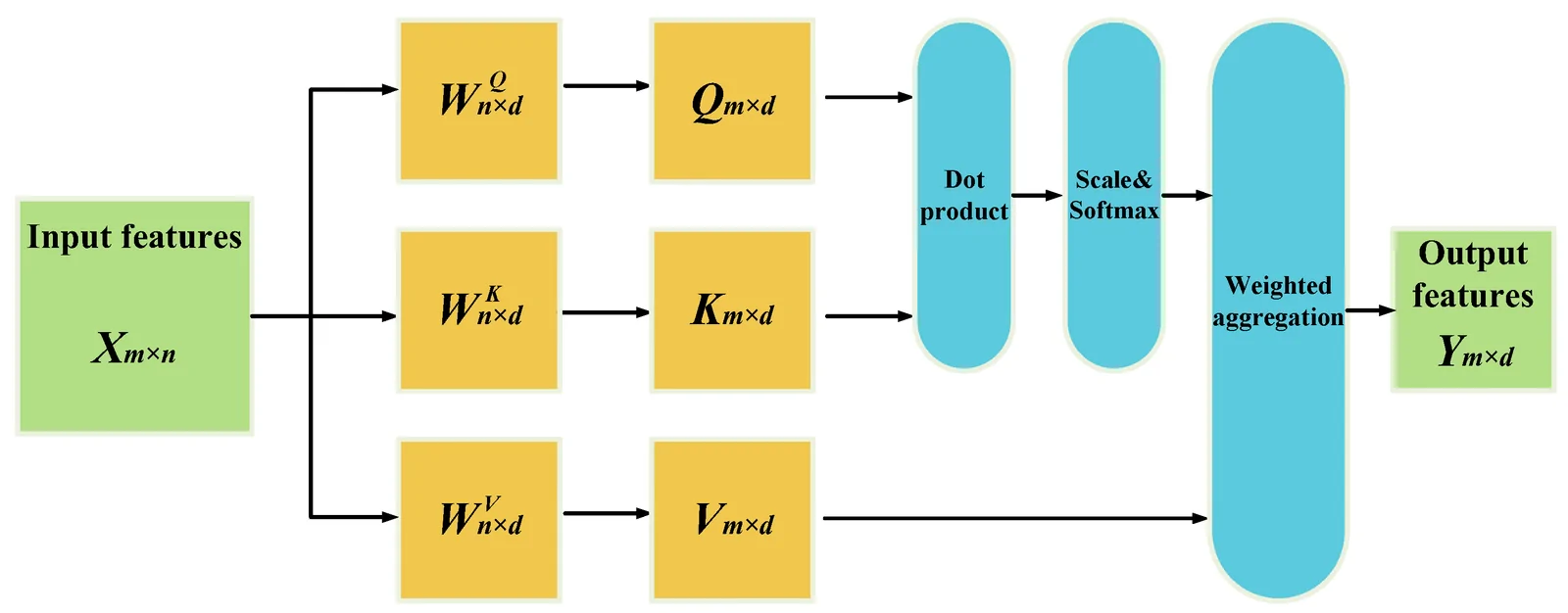
QKV computation in the attention mechanism.

Once the vibration features are extracted from the measured signals, further feature refinement is required because the resulting feature representation may contain redundant and noise-related components. With the rapid development of deep learning, the attention mechanism has emerged as an essential technique and has been widely applied in various fields, including natural language processing, statistical learning, image analysis, and speech recognition [30]. By adaptively assigning different weights to feature components, the attention mechanism enables neural networks to emphasize discriminative information while suppressing irrelevant features, thereby improving feature representation capability and information utilization efficiency. The basic structural framework of the attention mechanism in neural networks is illustrated in Figure 2.

Given an input feature matrix $X_{m\times n}$, the attention mechanism dynamically evaluates the importance of different feature components by modeling the dependencies among features. Through learning inter-feature correlations, the network can adaptively focus on feature representations that are highly relevant to fault characteristics. The attention operation can be formulated as follows

(3) $$Attention(Q,K,V)=\mathrm{Softmax}(\frac{QK^{T}}{\sqrt{d}})V$$

where $Q\epsilon R^{m\times d}$, $K\epsilon R^{m\times d}$, and $V\epsilon R^{m\times d}$ denote the query, key, and value matrices, respectively. Specifically, Q represents the query features used to evaluate feature relevance, K encodes the dependencies among different features, and V provides the feature representations for weighted aggregation. The parameter d denotes the feature dimension and is introduced to scale the dot-product attention scores. As illustrated in Figure 2, the dot-product operation first computes the similarity matrix $QK^{T}$. The similarity scores are then scaled by $\sqrt{d}$ and normalized using the Softmax function to obtain the attention weight matrix. Finally, the attention weights are multiplied by V in the weighted aggregation step to obtain the output feature representation. The query, key, and value matrices are obtained by linearly projecting the input features using learnable weight matrices, which can be expressed as

(4) $$Q=XW^{Q},\;\;K=XW^{K},\;\;V=XW^{V}$$

where $X\epsilon R^{m\times n}$ denotes the input feature matrix, $W^{Q}$, $W^{K}$ and $W^{V}\epsilon R^{n\times d}$ represent the learnable projection matrices for generating the query, key, and value representations, respectively.

#### 2.2.2. Residual Network Architecture

Although conventional deep learning architectures exhibit powerful capabilities in automatic feature extraction, increasing network depth often introduces optimization difficulties, such as gradient vanishing, gradient explosion, and network degradation, which hinder further performance improvements of deeper models. To address these issues, He et al. [31] proposed the residual network (ResNet), which introduces shortcut connections through identity mapping to facilitate information propagation across network layers. By adopting residual learning, ResNet improves the optimization of deep architectures and enables the extraction of more discriminative and robust feature representations.

**Figure 3:**
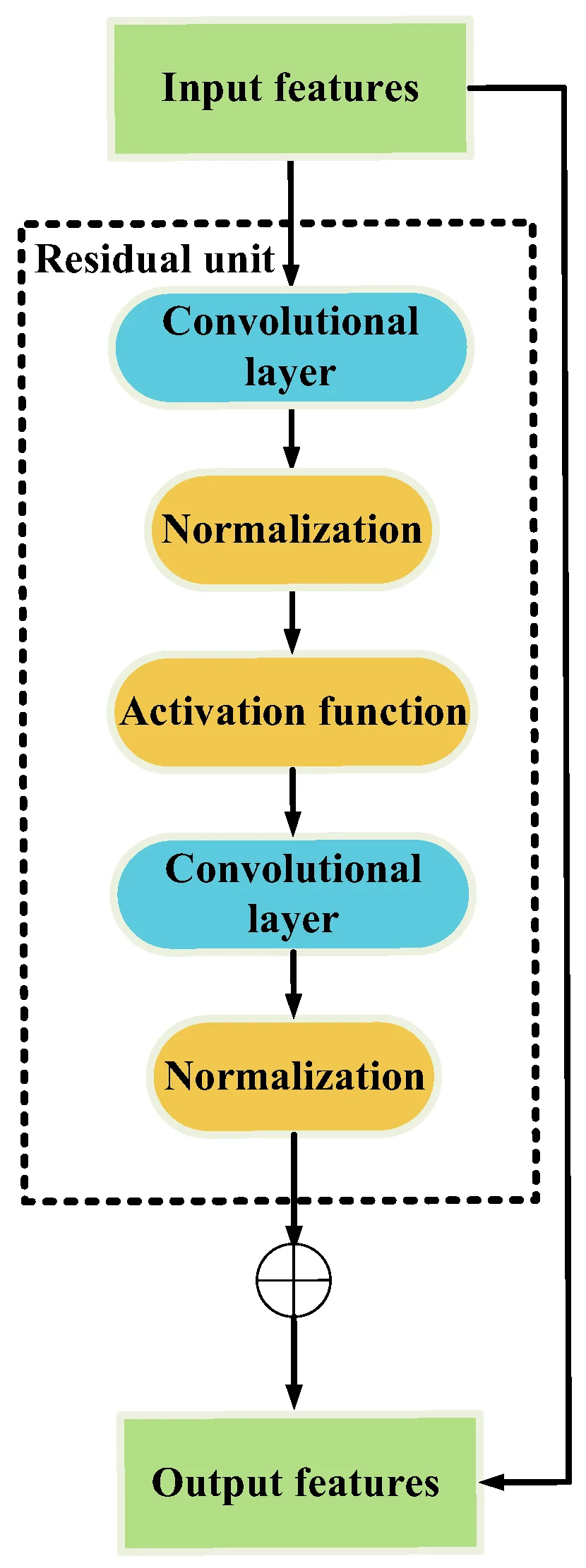
Residual network architecture.

The basic building block of ResNet is the residual unit, and its architecture is illustrated in Figure 3. Unlike traditional feedforward neural networks that directly learn the nonlinear mapping between input and output, the residual unit reformulates the learning objective by introducing a shortcut connection and optimizing the residual function. Specifically, instead of directly fitting the original mapping H(X), ResNet learns the residual mapping $F\left(X\right)=H\left(X\right)-X$, and the output of the residual unit can be expressed as

(5) Y=F(X)+X

where X denotes the input feature and $F\left(X\right)$ represents the residual mapping learned by the convolutional layers. The key advantage of the residual architecture is that it simplifies the optimization process by converting the original mapping learning problem into residual function learning. When deep convolutional layers encounter difficulties in learning an effective mapping, the residual branch can approximate zero, allowing the overall transformation to approach identity mapping. This mechanism facilitates gradient propagation through shortcut connections, alleviates the degradation problem of deep networks, and improves the optimization stability and trainability of deep architectures.

### 2.3. Multi-Kernel Domain Adaptation for Cross-Domain Fault Identification

Although deep learning-based diagnostic algorithms have achieved remarkable progress in mechanical fault identification, cross-domain recognition remains challenging because of significant discrepancies between different operating conditions. In this study, the cross-domain fault identification problem is formulated entirely based on experimentally measured vibration signals. Different rotational speeds and ball screw specimens are regarded as different domains because they induce changes in the statistical distributions of vibration features. Such domain discrepancies can reduce the generalization capability of conventional fault identification models trained under a single operating condition. Therefore, domain adaptation is introduced to reduce the distribution discrepancy between source and target domains while preserving fault-discriminative information.

In general, vibration signal data collected under the same operating condition can be regarded as belonging to a domain D, which consists of a feature space X and a marginal probability distribution P(x), i.e.,

(6) $$D=\left\{X,P(x)\right\}$$

where $x=\left\{x_{1},x_{2},\ldots ,x_{n}\right\}\in X$ represents the vibration samples collected from the corresponding domain, and *n* denotes the dimensionality of the feature space. Based on domain *D*, a learning task *T* can be defined as

(7) $$T=\left\{Y,f\right\}$$

where *Y* denotes the label space and *f* represents the prediction function learned from the feature space *X* and corresponding labels *Y*. Accordingly, transfer learning aims to improve the prediction capability of the target task $T_{t}$ in an unknown target domain $D_{t}$ by leveraging knowledge from a labeled source domain $D_{s}$ and its corresponding task $T_{s}$, where $D_{s}\neq D_{t}$ or $T_{s}\neq T_{t}$. Specifically, the objective is to learn an optimal prediction function $f_{t}$ for the target domain by transferring useful knowledge from the source domain and task.

In this study, the cross-domain fault identification problem of ball screw feed systems is reformulated based on the definition of transfer learning. The labeled source domain is defined as $D_{s}^{i}=\left\{X_{i},Y_{i}\right\}$, where vibration samples $X_{i}$ and their corresponding fault labels $Y_{i}$ are collected under known operating conditions. The target domain is defined as $D_{t}=\left\{X\right\}$, consisting of unlabeled vibration samples collected from target operating conditions or different ball screw specimens. Since all datasets are experimentally measured, the domain shifts considered in this study mainly originate from variations in rotational speed and ball screw specimen characteristics.

**Figure 4:**
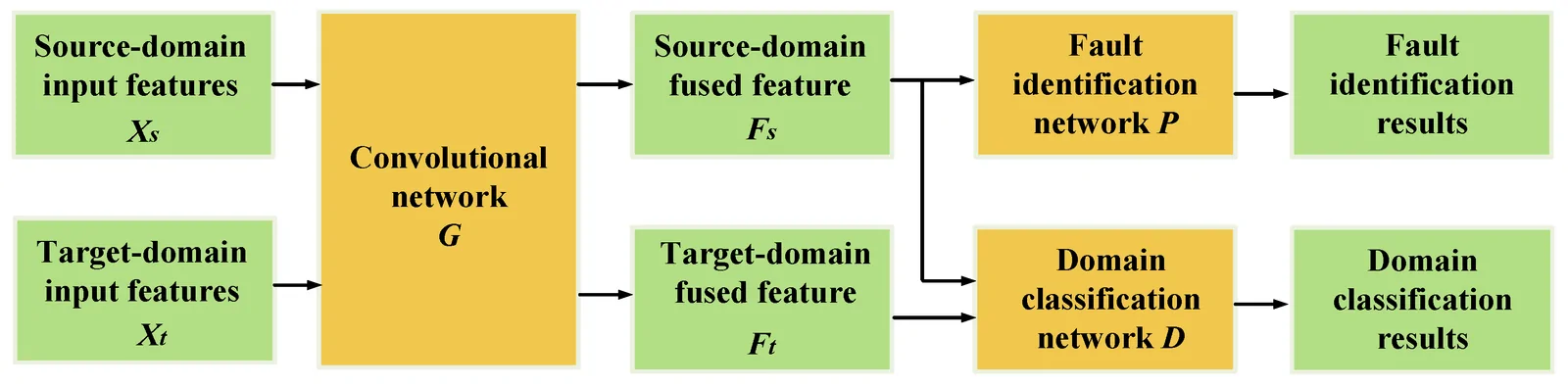
General cross-domain fault identification framework.

The general cross-domain fault identification framework proposed in this study is illustrated in Figure 4. The framework is trained using labeled source-domain data and unlabeled target-domain data. A shared feature extraction and fusion network *G* is first employed to transform the input vibration feature representations from both domains into compact fused feature representations, denoted by *F_s_* and *F_t_* for the source and target domains, respectively. The source-domain fused feature representation *F_s_* is subsequently fed into the fault classification network *P*, through which the source-domain fault classification loss *L_P_* is evaluated. Meanwhile, the fused feature representations *F_s_* and *F_t_* are jointly used for cross-domain distribution alignment. Specifically, an adversarial domain discrimination mechanism is employed to encourage the feature extraction and fusion network *G* to learn domain-invariant representations, while MK-MMD is introduced to explicitly quantify and minimize the distribution discrepancy between the source- and target-domain feature representations. Through the joint optimization of source-domain fault classification and cross-domain distribution alignment, the proposed framework aims to learn feature representations that preserve fault-discriminative information while reducing domain-specific variations.

The maximum mean discrepancy (MMD) is introduced to explicitly quantify the distribution discrepancy between the source and target domains. As a nonparametric distributional distance measure, MMD maps samples into a reproducing kernel Hilbert space (RKHS) through kernel embedding and evaluates the discrepancy between the corresponding mean embeddings. The MMD is defined in Equation (8).

(8) $$MMD(X_{i},X_{j})={\left\|\frac{1}{n_{i}}\sum_{p=1}^{n_{i}}\phi (x_{p}^{i})-\frac{1}{n_{j}}\sum_{q=1}^{n_{j}}\phi (x_{q}^{j})\right\|}_{H}$$

where *X_i_* and *X_j_* denote the feature distributions under two different operating conditions, while *x^i^* and *x^j^* represent individual samples drawn from the corresponding distributions. *n* indicates the number of samples in each operating condition. *H* represents the RKHS, and *ϕ*(·) denotes the nonlinear feature mapping function that transforms the original data into the RKHS. With a properly selected kernel function, the inner product between two mapped samples in the RKHS can be expressed as

(9) $$\langle \phi (x_{i}),\phi (x_{j})\rangle =k(x_{i},x_{j})$$

where the kernel function *k*(·,·) can be implemented using different kernel strategies, including linear, polynomial, and Gaussian kernels, to characterize the nonlinear relationships between samples. The corresponding mathematical expressions are formulated as follows

(10) $$k(x_{i},x_{j})=\left\{\begin{array}{l} \langle x_{i},x_{j}\rangle \;\;\;\;\;\;\;\;\;\;\;\;\;\;\;\;\;\;\;\mathrm{linear}\;\mathrm{kernel}\\ {\langle x_{i},x_{j}\rangle }^{d}\;\;\;\;\;\;\;\;\;\;\;\;\;\;\;\;\;\;\mathrm{polynomial}\;\mathrm{kernel}\\ \exp\left(-{\left\|x_{i}-x_{j}\right\|}_{2}^{2}/2\sigma^{2}\right)\;\;\;\;\;\;\;\;\;\mathrm{Gaussian}\;\mathrm{kernel} \end{array}\right.$$

For cross-domain fault identification, the choice of kernel function directly affects the accuracy of domain distribution characterization, because different kernel functions correspond to different RKHSs and thus lead to different domain partitioning results. To improve the capability of the model to characterize the distribution discrepancy between the source and target domains, a multi-kernel strategy is introduced to replace the conventional single-kernel approach. Specifically, MK-MMD is employed as an alternative to conventional MMD and is defined in Equation (11).

(11) $$\mathrm{MK}\text{-}\mathrm{MMD}(F_{s},F_{t})=\sum_{l=1}^{K}\beta_{l}{\mathrm{MMD}}_{k_{l}}(F_{s},F_{t})$$

where K denotes the number of employed kernel functions, $k_{l}$ represents the *l*-th kernel function, and $\beta_{l}$ denotes its corresponding weight. By integrating distribution discrepancies measured in multiple RKHSs, MK-MMD reduces the dependence on a single kernel and provides a more comprehensive characterization of cross-domain distribution discrepancies.

## 3. Design of Fault Diagnosis Model

### 3.1. MK-DANN Network Model

**Figure 5:**
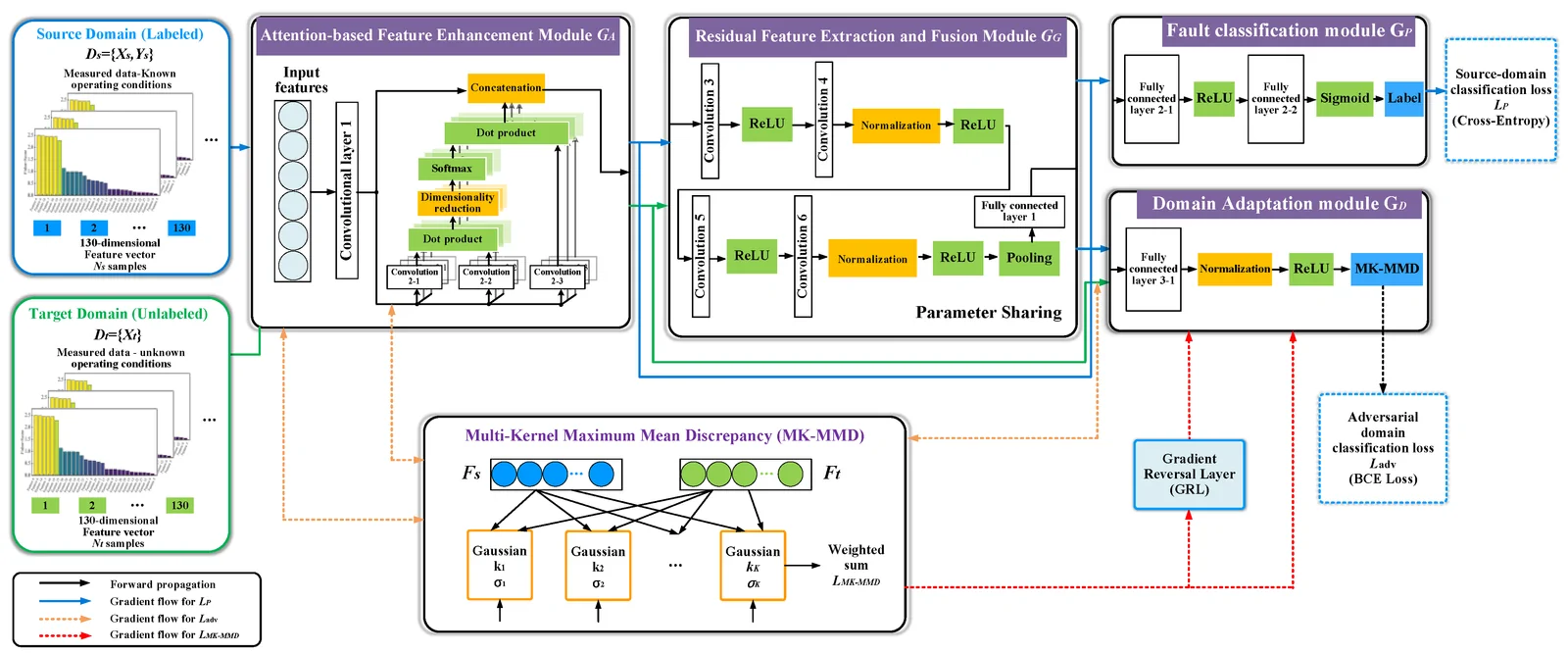
Architecture of the proposed FE-MK-DANN.

Based on the above convolutional neural network framework, a feature-enhanced multi-kernel domain adversarial neural network (FE-MK-DANN) is proposed for cross-domain fault identification of ball screw feed systems. The overall architecture of the proposed model is illustrated in Figure 5. The model takes the extracted vibration feature representations as inputs and simultaneously performs fault classification and domain adaptation. Specifically, the FE-MK-DANN consists of four major modules: an attention-based feature enhancement module *G_A_* for adaptive feature weighting, a residual feature extraction and fusion module *G_G_* for hierarchical representation learning, a fault classification module *G_P_* for fault decision-making, and a domain adaptation module *G_D_* for adversarial domain discrimination. In addition, MK-MMD is introduced to explicitly measure the distribution discrepancy between the fused source- and target-domain feature representations.

In the attention-based feature enhancement module *G_A_*, vibration feature representations from both the source and target domains are first processed by convolutional layers to obtain preliminary feature representations. Zero-padding is employed to preserve the dimensionality of the feature representation. Subsequently, a self-attention mechanism adaptively recalibrates the importance of different feature components. By modeling the interactions among feature components, the attention mechanism emphasizes informative and fault-discriminative representations while suppressing redundant and noise-related components. The enhanced representations are subsequently fed into the residual feature extraction and fusion module *G_G_*, where hierarchical features are learned through successive convolution, normalization, activation, and residual operations. Residual connections facilitate information and gradient propagation and alleviate the degradation problem associated with increasing network depth. The resulting feature maps are aggregated through average pooling and fully connected layers to obtain compact fused feature representations.

The fused source-domain features are subsequently fed into the fault classification module *G_P_* to predict the corresponding fault categories. Meanwhile, the fused source- and target-domain features are jointly introduced into the domain adaptation module *G_D_* for adversarial domain discrimination. In parallel, MK-MMD is directly calculated using the fused source- and target-domain features to quantify their distribution discrepancy from multiple RKHSs. The joint optimization of fault classification, adversarial domain adaptation, and MK-MMD-based distribution alignment enables the network to learn feature representations that are both fault-discriminative and domain-invariant.

### 3.2. Model Training Strategy

To improve the cross-domain fault identification capability of the proposed FE-MK-DANN, the model is trained by jointly optimizing fault classification and domain adaptation objectives. The training process involves three complementary loss terms: the source-domain fault classification loss *L_P_*, the adversarial domain classification loss *L*_adv_, and the multi-kernel distribution discrepancy loss *L*_MK−MMD_. The fault classification loss preserves fault-discriminative information, whereas the latter two objectives reduce domain-specific variations between the source and target domains.

The fault prediction loss *L_P_* is designed to optimize the feature enhancement network *G_A_*, feature fusion network *G_G_*, and fault classification network *G_P_* in the proposed FE-MK-DANN model. By minimizing *L_P_*, the network is guided to adaptively learn the mapping relationship between input feature representations and corresponding fault labels. The fault classification loss *L_P_* is calculated using the cross-entropy function based on the labeled source-domain samples.

(12) $$L_{P}=-\frac{1}{N_{s}}\sum_{i=1}^{N_{s}}\sum_{c=1}^{C}y_{ic}\log{\hat{y}}_{ic}$$

where *N_s_* denotes the number of labeled source-domain samples, *C* is the number of fault categories, $y_{ic}$ represents the ground-truth label of the *i*-th source-domain sample for category *c*, and ${\hat{y}}_{ic}$ denotes the corresponding predicted probability, which can be expressed as Equation (13).

(13) $${\hat{y}}_{i}=G_{P}(F_{i};\theta_{P})$$

The MK-MMD loss is employed to explicitly measure the distribution discrepancy between the fused source-domain features $F_{s}$ and target-domain features $F_{t}$. Unlike conventional single-kernel MMD, MK-MMD integrates multiple kernel functions with different characteristics to characterize the distribution discrepancy from multiple RKHSs. Accordingly, the MK-MMD loss is defined as

(14) $$L_{\mathrm{MK}\text{-}\mathrm{MMD}}=\sum_{l=1}^{K}\beta_{l}{\mathrm{MMD}}_{k_{l}}(F_{s},F_{t})$$

where *K* denotes the number of employed kernel functions, $k_{l}$ represents the *l*-th kernel function, and $\beta_{l}$ denotes its corresponding weight. By combining multiple kernel-induced distribution discrepancies, MK-MMD reduces the dependence of domain alignment on a single RKHS and provides a more robust characterization of cross-domain feature discrepancies.

The adversarial domain classification loss *L*_adv_ is introduced to distinguish the source and target domains. Let the source-domain samples be assigned a domain label of 1 and the target-domain samples a domain label of 0. The domain classification loss is defined using binary cross-entropy as

(15) $$L_{\mathrm{adv}}=-\frac{1}{N_{s}+N_{t}}\left[\sum_{i=1}^{N_{s}}\log{\hat{d}}_{i}^{s}+\sum_{j=1}^{N_{t}}\log\left(1-{\hat{d}}_{j}^{t}\right)\right]$$

where $N_{s}$ and $N_{t}$ denote the numbers of source- and target-domain samples, respectively, and ${\hat{d}}_{i}^{s}$ and ${\hat{d}}_{j}^{t}$ represent the predicted probabilities that the corresponding samples belong to the source domain, which can be expressed as Equation (16).

(16) $${\hat{d}}_{i}^{s}=G_{D}\left(F_{i}^{s};\theta_{D}\right),\;{\hat{d}}_{j}^{t}=G_{D}\left(F_{j}^{t};\theta_{D}\right)$$

Based on the above adversarial optimization strategy, the optimization objective of the FE-MK-DANN network is defined to simultaneously preserve fault-discriminative information and reduce the distribution discrepancy between the source and target domains. By integrating the fault prediction loss, the MK-MMD loss, and the adversarial domain-classification loss described in Equations (12), (14), and (15), the overall loss function is formulated as

(17) $$L_{FE}=L_{P}+\lambda_{1}L_{\mathrm{MK}\text{-}\mathrm{MMD}}+\lambda_{2}\left(p\right)L_{\mathrm{adv}}$$

where $\lambda_{1}$ and $\lambda_{2}$ are the trade-off coefficients controlling the contributions of the MK-MMD loss and adversarial domain-classification loss, respectively. In this study, $\lambda_{1}$ is fixed at 1 throughout the domain-adaptation stage. The adversarial coefficient $\lambda_{2}$ is progressively increased following the scheduling strategy of Ganin et al. [32], as expressed in Equation (18).

(18) $$\lambda_{2}\left(p\right)=2/\left(1+\exp\left(-10p\right)\right)-1$$

where $p\in \left[0,1\right]$ denotes the normalized progress of the domain-adaptation stage. Thus, the adversarial objective is gradually strengthened as training proceeds, which helps avoid excessively strong domain alignment during the early stage of feature learning.

The parameters of FE-MK-DANN are optimized through adversarial domain adaptation. The domain discriminator *G_D_* minimizes the weighted domain-classification loss $\lambda_{2}(p)L_{adv}$ to distinguish source- and target-domain features. For the feature enhancement module *G_A_* and the feature fusion module *G_G_*, the gradient associated with $\lambda_{2}(p)L_{adv}$ is reversed by the gradient reversal layer (GRL) during backpropagation, thereby encouraging the extraction of domain-invariant feature representations. The fault classifier *G_P_* is optimized by the source-domain classification loss. Therefore, the GRL is the only mechanism used to reverse the adversarial gradient, and no additional negative sign is introduced before $L_{adv}$ in Equation (17).

Accordingly, the optimization objectives of the feature-learning modules and the domain discriminator can be expressed in Equation (19).

(19) $$\left\{\begin{array}{l} (\theta_{A}^{*},\theta_{G}^{*},\theta_{P}^{*})=\underset{\theta_{A},\theta_{G},\theta_{P}}{\arg\min}\left[L_{P}+\lambda_{1}L_{\mathrm{MK}\text{-}\mathrm{MMD}}-\lambda_{2}\left(p\right)L_{\mathrm{adv}}\right]\\ \theta_{D}^{*}=\underset{\theta_{D}}{\arg\min}\;\lambda_{2}\left(p\right)L_{\mathrm{adv}} \end{array}\right.$$

where $\theta_{A},\theta_{G},\theta_{P}$, and $\theta_{D}$ denote the trainable parameters of *G_A_*, *G_G_*, *G_P_*, and *G_D_*, respectively. The negative sign in the first optimization objective represents the adversarial effect induced by the GRL on the feature-learning modules and is not an additional negative term in the scalar loss of Equation (17).

During backpropagation, the parameter updates are therefore given in Equation (20).

(20) $$\left\{\begin{array}{l} \theta_{A}\leftarrow \theta_{A}-\eta \left(\frac{\partial L_{P}}{\partial \theta_{A}}+\lambda_{1}\frac{\partial L_{\mathrm{MK}\text{-}\mathrm{MMD}}}{\partial \theta_{A}}-\lambda_{2}\left(p\right)\frac{\partial L_{\mathrm{adv}}}{\partial \theta_{A}}\right)\\ \theta_{G}\leftarrow \theta_{G}-\eta \left(\frac{\partial L_{P}}{\partial \theta_{G}}+\lambda_{1}\frac{\partial L_{\mathrm{MK}\text{-}\mathrm{MMD}}}{\partial \theta_{G}}-\lambda_{2}\left(p\right)\frac{\partial L_{\mathrm{adv}}}{\partial \theta_{G}}\right)\\ \theta_{P}\leftarrow \theta_{P}-\eta \frac{\partial L_{P}}{\partial \theta_{P}}\\ \theta_{D}\leftarrow \theta_{D}-\eta \lambda_{2}\left(p\right)\frac{\partial L_{\mathrm{adv}}}{\partial \theta_{D}} \end{array}\right.$$

where *η* denotes the learning rate. In this formulation, the domain discriminator minimizes $L_{adv}$, whereas the GRL reverses only the adversarial gradient propagated to *G_A_* and *G_G_*.

## 4. Experimental Analysis and Discussion

### 4.1. Test Description

**Figure 6:**
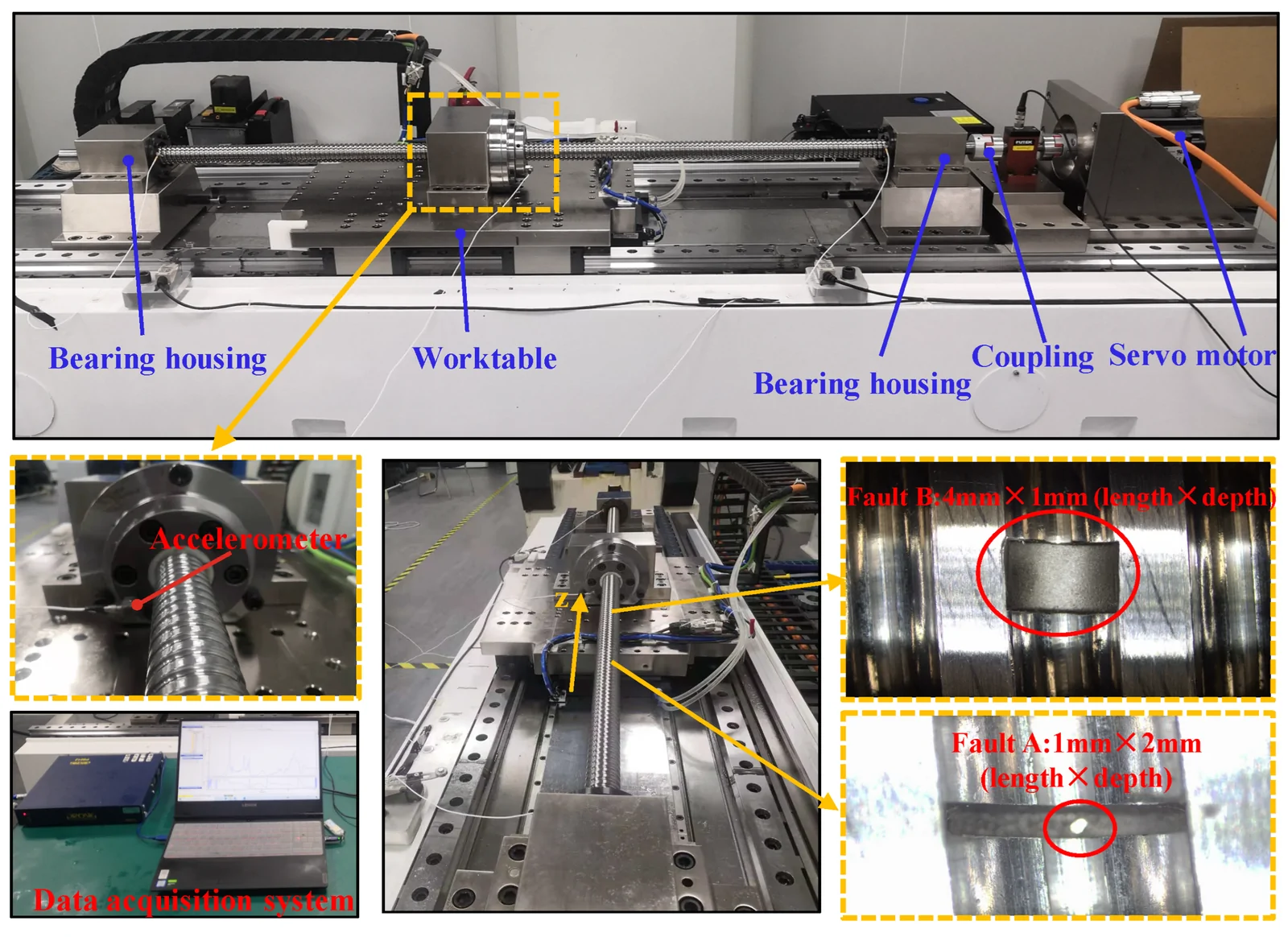
Linear feed test platform and ball screw with artificial spalling defects.

To verify the proposed method, a series of spalling fault identification experiments on ball screws are performed. The experimental tests are conducted on the vibration test platform presented in Figure 6, and the detailed specifications of the test rig are provided in Table 2. Two 4010-type double-nut internal circulation ball screws (Shaanxi Hanjiang Machine Tool Co., Ltd., Hanzhong, China) are selected as experimental specimens, with their main geometric and structural parameters summarized in Table 3. The initial preload values of the two specimens are 2660 N and 2380 N, respectively. Prior to the fault experiments, the lead errors of the ball screw specimens are measured using the measurement system shown in Figure 6. Subsequently, after installation into the feed drive system, the assembly-induced installation errors of the ball screws are measured to ensure accurate characterization of the initial operating conditions.

Two artificial spalling defects are introduced at the same locations on the two ball screws to simulate typical service-induced failures, as illustrated in Figure 6. The first defect (fault A) is located 160 mm from the starting point of the stroke, and the second defect (fault B) is 200 mm away from fault A. The overall length of the double-nut assembly is 130 mm. During the experiments, vibration responses are measured under six rotational speeds of 50, 100, 150, 200, 250, and 300 rpm. A Dytran 3255A3 accelerometer (Dytran Instruments, Inc., Chatsworth, CA, USA) is installed at the nut flange to acquire the axial vibration response of the nut along the *z*-axis. The vibration signals are continuously acquired, monitored, and stored using a Prosig P8012 data acquisition system (Prosig Ltd., Great Torrington, UK) with a sampling frequency of 25.6 kHz, which enables sufficient capture of the characteristic vibration components associated with the spalling defects.

### 4.2. Signal Preprocessing and Feature Extraction

**Figure 7:**
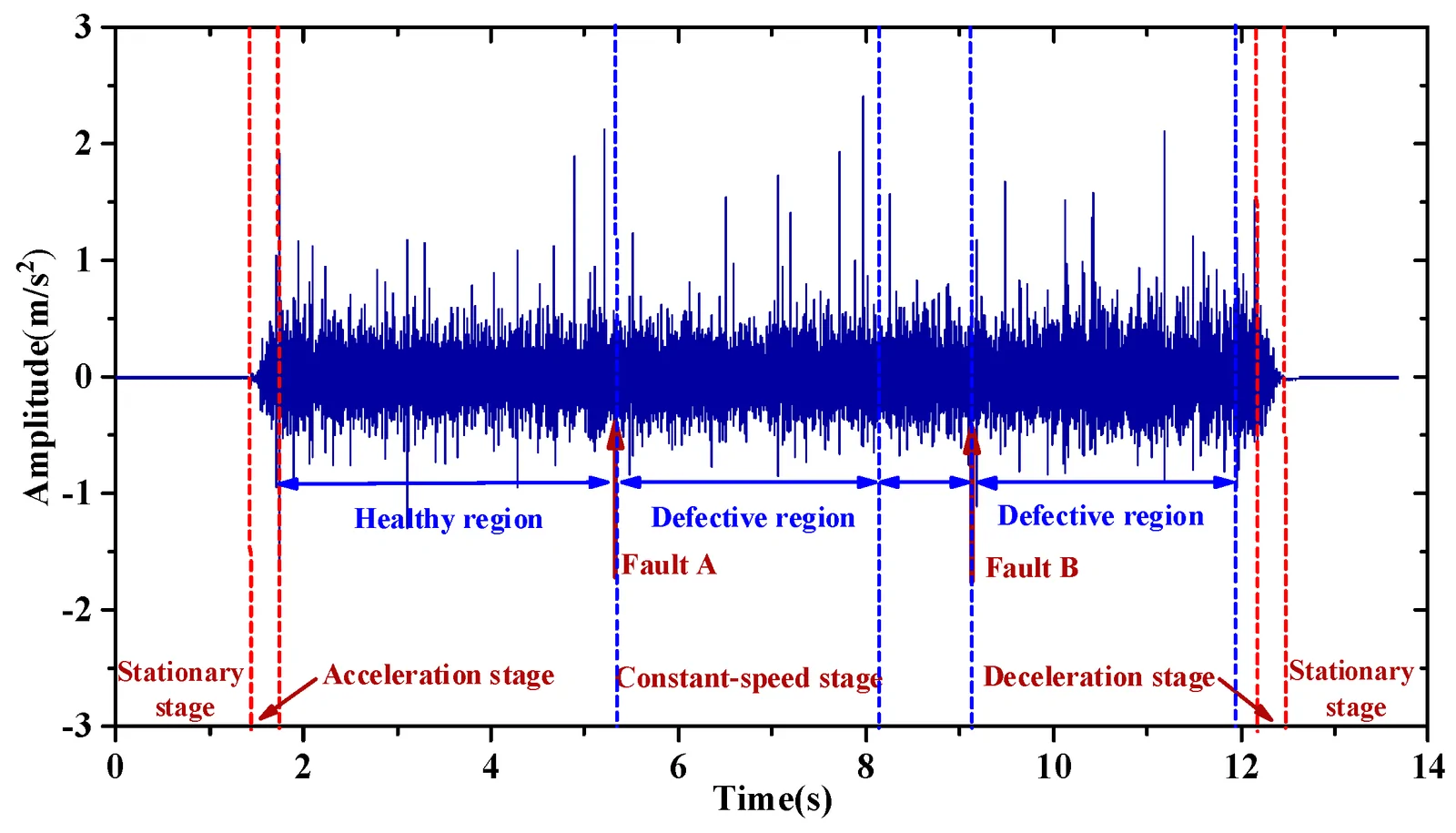
Raw vibration time-domain waveform of specimen 1 at 100 rpm.

Due to the presence of substantial background noise in the measured signals, signal preprocessing is first performed to improve the quality of the vibration data and the reliability of subsequent feature extraction. Figure 7 presents the time-domain waveform of the raw vibration signal acquired from specimen 1 at a rotational speed of 100 rpm. The complete signal can be divided into five stages, namely the initial stationary stage, acceleration stage, constant-speed stage, deceleration stage, and final stationary stage. Since the acceleration and deceleration stages correspond to transient operating conditions, only the constant-speed stage is retained for subsequent feature extraction and fault identification to ensure consistency among the experimental samples.

**Figure 8:**
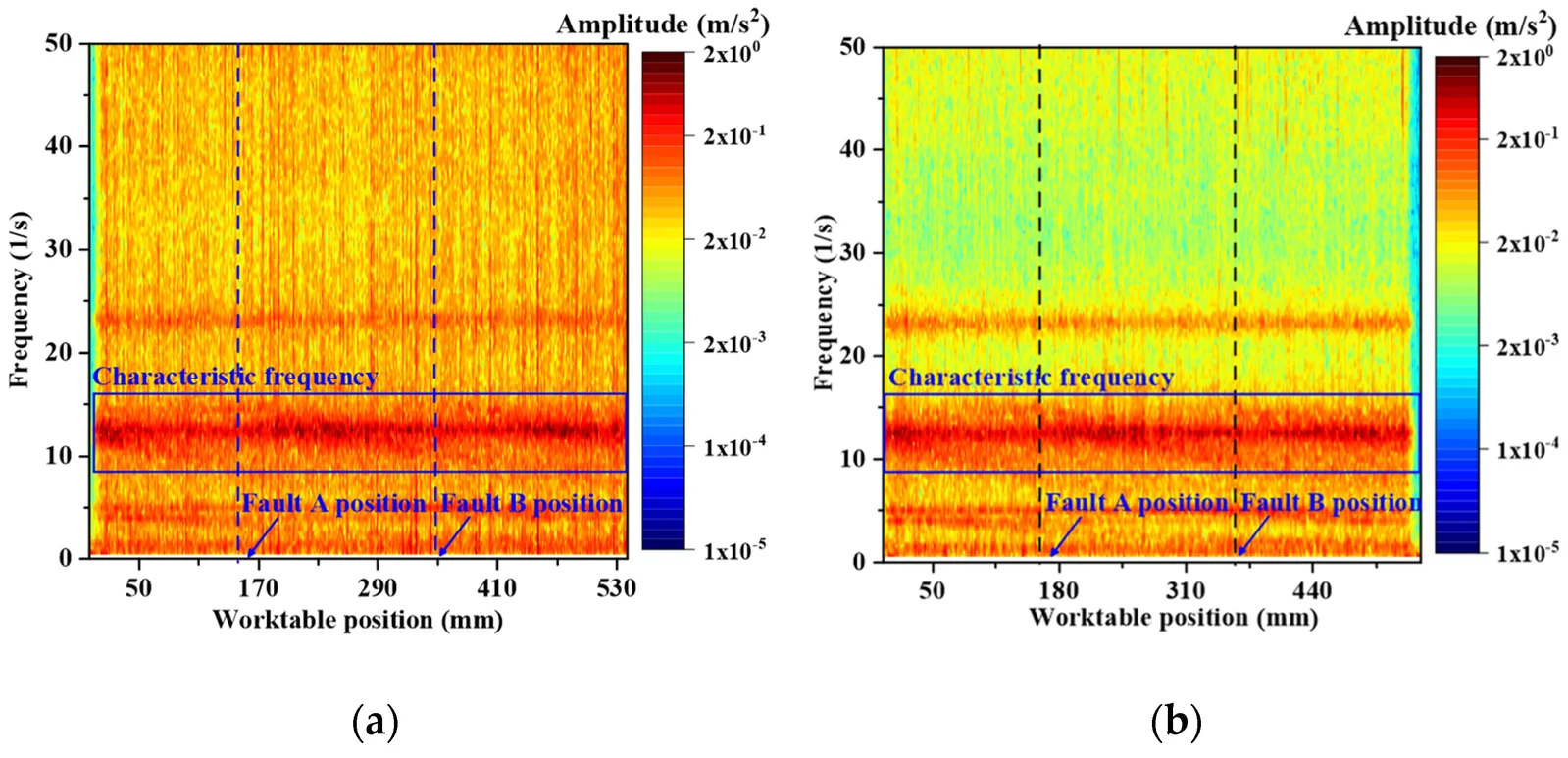
Spatial-frequency representation of the raw vibration signals: (**a**) Specimen 1; (**b**) Specimen 2.

During the constant-speed stage, the nut successively passes through spalling positions A and B. However, the fault-related differences cannot be reliably identified from the raw time-domain signals because the vibration responses associated with defective and healthy regions are strongly masked by background noise. In addition, the spatial locations of the spalling defects cannot be directly determined from the raw vibration waveform. Therefore, the measured signals are transformed into the spatial-frequency domain to characterize the spatial distribution of the measured vibration responses. The corresponding spatial-frequency representations of the two ball screw specimens are shown in Figure 8. Although the transformation provides spatial-frequency information, the defective regions remain difficult to distinguish from healthy regions. Moreover, the frequency components associated with the measured fault responses and their harmonics are substantially contaminated by broadband noise, making it difficult to obtain sufficiently discriminative features. Although the amplitudes in the defective regions are generally higher than those in the healthy regions, their spatial distributions exhibit considerable variability. These observations indicate that the spatial-frequency representation alone is insufficient for reliable fault localization and identification.

A set of time-domain and frequency-domain statistical features is subsequently extracted from the measured vibration signals, with the detailed definitions provided in Table A1 and Table A2 of Appendix A. The signals are transformed into the frequency domain using the fast Fourier transform (FFT), and the amplitudes of the first 100 frequency bins are retained. The extracted features are then normalized using the Robust Z-score method. For each cross-domain task, the median and interquartile range (IQR) are estimated exclusively from the source-domain training samples and applied unchanged to the target-domain samples. These statistics are subsequently fixed and applied unchanged to both the source-domain and target-domain samples, thereby preventing target-domain information from entering the preprocessing procedure. Subsequently, Uniform Manifold Approximation and Projection (UMAP) [33] is employed to project the high-dimensional feature representations into a low-dimensional space for visualizing their distribution characteristics under different ball screw specimens and rotational speeds.

**Figure 9:**
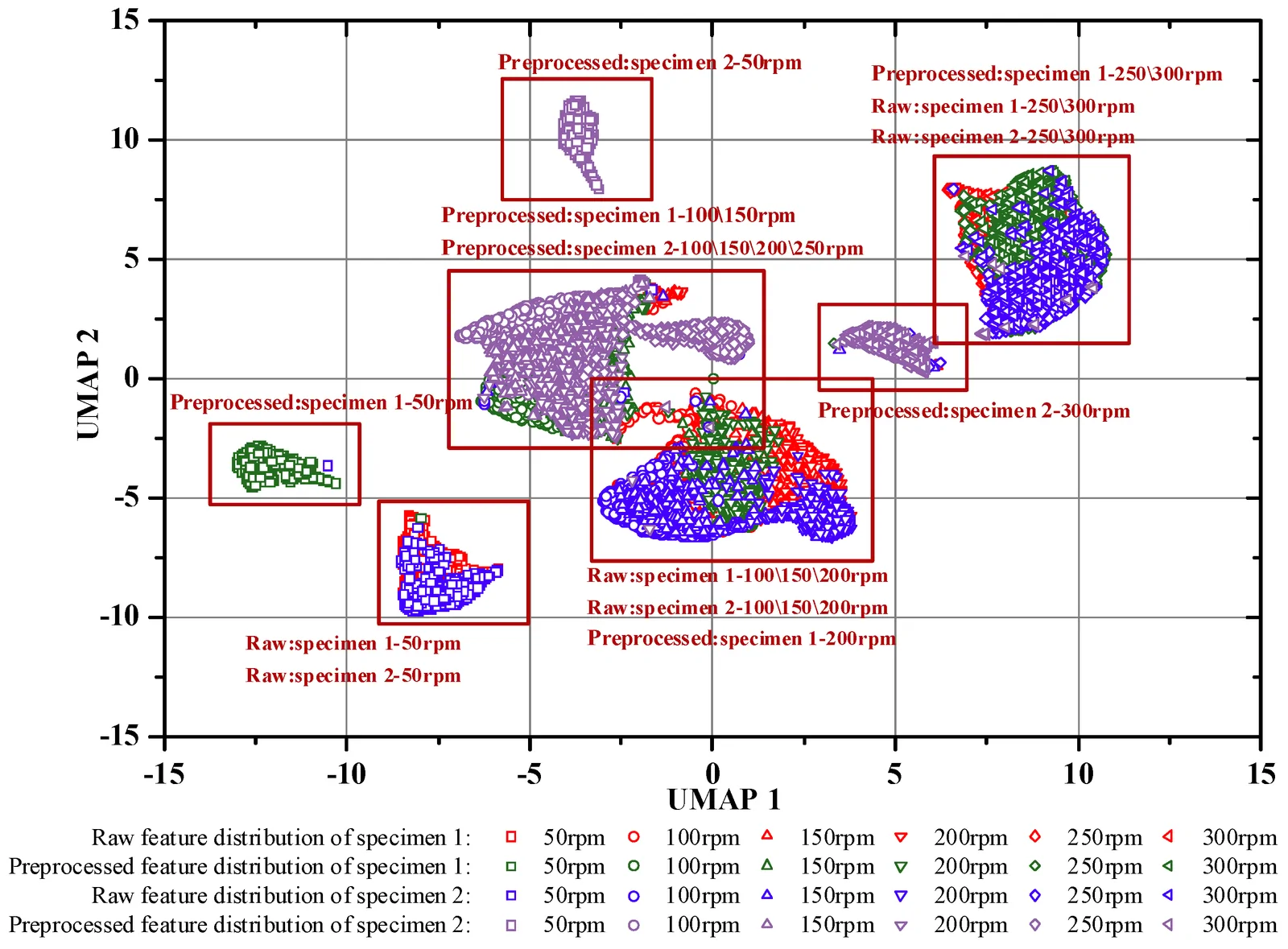
UMAP visualization of vibration feature distributions before and after signal preprocessing.

Figure 9 presents the UMAP visualization of the vibration feature distributions under different operating conditions. Before signal preprocessing, the feature distributions of the two ball screw specimens exhibit substantial overlap, and samples collected at different rotational speeds are not clearly separated. This observation indicates that noise and irrelevant signal components may obscure the distributional differences among different specimens and operating conditions. After signal preprocessing, the feature distributions become more compact and structured, with reduced intra-domain dispersion and clearer distributional characteristics across operating conditions. These results indicate that the preprocessing procedure improves the quality and discriminability of the extracted vibration features, providing a more suitable input representation for subsequent cross-domain fault identification using the proposed FE-MK-DANN.

### 4.3. Dataset Description and Cross-Domain Task Formulation

The training datasets are constructed from experimentally measured vibration signals collected using the ball screw feed system test platform described in Section 4.1. Different domains are generated by varying operating speeds and ball screw specimens, thereby simulating practical domain shifts in industrial fault diagnosis scenarios. According to the locations of the artificially introduced spalling defects, the fault samples collected in experiments are divided into two categories. The defect at position A is regarded as a level-I spalling fault, whereas the defect at position B is considered a level-II spalling fault. Accordingly, the single-specimen fault identification task includes three health conditions, namely healthy condition, level-I spalling fault, and level-II spalling fault. Six rotational speeds (50, 100, 150, 200, 250, and 300 rpm) are configured for the experimental datasets. As a result, the datasets constructed from the two ball screw specimens comprise 12 operating conditions in total.

The extracted statistical feature vectors, together with the selected frequency-domain amplitude components, are used as the network inputs. Specifically, each segmented time-domain vibration signal is transformed into the frequency domain using the fast Fourier transform (FFT), and the amplitudes corresponding to the first 100 frequency bins are retained. Since the FFT frequency bins are uniformly spaced, these 100 components represent consecutive and equally spaced frequency locations in the low-frequency region. The number of retained frequency bins was determined by examining the spectra of the source-domain training samples, which showed that the dominant vibration energy was concentrated within this low-frequency range. Importantly, this selection criterion was determined exclusively from the source-domain training data and was subsequently fixed for all target-domain samples; no target-domain information was used in determining either the number or the locations of the selected frequency bins.

To construct spatially distributed samples, the vibration signals are segmented using non-overlapping spatial windows with a length of 1 mm, generating 540 spatial samples for each operating condition. Although the windows are non-overlapping, neighboring samples may exhibit correlated vibration characteristics because they are acquired from spatially adjacent positions. Therefore, the resulting samples are not assumed to be statistically independent, and the potential influence of spatial correlation on random train/test splitting is further evaluated using a block-wise splitting strategy in Section 4.4. The complete dataset consists of 12 operating conditions, with 540 samples generated from each operating condition. Specifically, the dataset contains 320 normal samples, 110 level-I spalling fault samples, and 110 level-II spalling fault samples. Each segmented vibration sample represents the measured local vibration response of the ball screw feed system at a specific spatial position and is assigned the corresponding fault label. Each vibration sample is represented by a 130-dimensional feature vector consisting of 16 time-domain statistical features, 14 frequency-domain statistical features, and 100 frequency-bin amplitude components. To reduce the influence of feature-scale variations, all input features are normalized using the Robust Z-score method. For each cross-domain task, the median and IQR are estimated exclusively from the source-domain training samples and subsequently applied unchanged to the target-domain samples. The detailed dataset configuration is summarized in Table 4.

The cross-domain learning strategy considered in this study follows a transductive unsupervised domain adaptation setting. During adaptation, labeled source-domain samples and unlabeled samples from the specific target domain are jointly used for feature alignment, while no target-domain fault labels participate in training. Therefore, deployment to a new target domain requires the acquisition of unlabeled target-domain measurements and an additional adaptation stage before diagnostic inference. The proposed FE-MK-DANN model is trained through two progressive stages. In the first stage, the model is pre-trained using labeled vibration samples collected from the source domain to establish the mapping relationship between vibration features and fault categories. After convergence, the learned parameters of the feature enhancement and feature fusion modules are retained and transferred to initialize the subsequent domain adaptation process. In the second stage, different source-target domain pairs are constructed according to various cross-speed and cross-specimen scenarios. During this stage, the labeled source-domain samples are used for fault classification, while the unlabeled target-domain samples participate only in domain adaptation. Through adversarial learning combined with MK-MMD-based distribution alignment, the feature distributions between source and target domains are reduced, enabling the model to extract domain-invariant fault representations and achieve reliable fault identification under previously unlabeled target operating conditions.

Each specimen-speed operating condition contains 540 samples. For each transfer task, all samples assigned to the source domain are used for source-domain training. The target-domain dataset contains 540 samples and is randomly divided once into two non-overlapping subsets using a fixed data-splitting seed of 42. Specifically, 270 samples (50%) are used as the unlabeled target adaptation set, while the remaining 270 samples (50%) are reserved exclusively as the target test set. During domain adaptation, the target adaptation samples participate only in adversarial domain alignment and MK-MMD-based distribution alignment and do not contribute to the classification loss. The target test set is not used during training or adaptation and is used only for final performance evaluation. To evaluate the influence of spatial correlation, an additional block-wise spatial splitting strategy was adopted. Since the fault conditions correspond to localized spatial regions, directly dividing the entire stroke into two halves could result in class imbalance or missing fault categories. Therefore, contiguous samples within each health-condition region were grouped into spatial blocks and assigned separately to the target adaptation and test sets.

The detailed settings of the constructed cross-domain tasks are listed in Table 5, including 12 different domain adaptation scenarios. Specifically, task $T_{A1}$ represents a speed-domain adaptation problem within the same ball screw specimen. In this task, all labeled samples from specimen 1 under the operating speeds of 100–300 rpm are defined as the source domain, whereas the samples collected from the same specimen at 50 rpm are considered the target domain. During adversarial training, all source-domain samples and half of the target-domain samples are simultaneously introduced into the network. The target-domain samples are exclusively used for domain alignment and do not contribute to the calculation of the classification loss. Task $T_{B1}$ represents a specimen-domain adaptation problem. In this task, all labeled samples from specimen 1 at 50 rpm are selected as the source domain, and the unlabeled samples from specimen 2 at the same rotational speed are used as the target domain. The subsequent adversarial adaptation procedure is consistent with that of task $T_{A1}$.

### 4.4. Results and Performance Analysis

To further verify the superiority of the proposed method, three representative deep learning-based fault diagnosis models are employed for comparison, including the conventional CNN without domain adaptation, the transfer convolutional neural network (TCNN) [34], and the domain adversarial neural network (DANN) [32]. These benchmark models cover both traditional feature learning without domain adaptation and transfer learning-based approaches, enabling a comprehensive evaluation of the domain adaptation capability of the proposed FE-MK-DANN framework. Common optimization hyperparameters were tuned using the same candidate ranges and tuning budget, whereas method-specific hyperparameters were optimized separately within predefined ranges. Hyperparameter selection was performed without access to target-domain test labels. The detailed network architecture and parameters are presented in Table 6. In the MK-MMD module, five Gaussian kernels with fixed bandwidths of 0.25, 0.5, 1, 2, and 4 are employed [35,36]. The kernel weights are fixed and equally assigned $\beta_{l}\;$= 1/5 rather than learned during training. The bandwidths are defined with respect to the normalized fused-feature space output by *G_G_*, on which the MK-MMD loss is computed. Specifically, the fused representations undergo Layer Normalization before distribution discrepancy measurement. The multiple bandwidths characterize distribution discrepancies at different scales in the normalized feature space, thereby reducing the sensitivity of domain alignment to a single kernel bandwidth.

The proposed FE-MK-DANN framework employs a two-stage training strategy for cross-domain fault identification. Initially, the model is pre-trained using labeled vibration data collected under the source operating condition. After obtaining stable fault-discriminative features, the model parameters are transferred to initialize domain adaptation for different cross-domain tasks, including cross-speed and cross-specimen scenarios. The entire training process contains 50 epochs with a batch size of 8. In the pre-training phase, the learning rate of the network is initialized as 0.005. During the subsequent adaptation phase with experimental fault data, a smaller learning rate of 0.0005 is assigned to the feature fusion module *G* to preserve the transferred feature representations, whereas the other modules are initialized with a learning rate of 0.003. The learning rate is reduced to 10^−4^ after 40 epochs. The complete training hyperparameters are provided in Table 7.

To avoid underestimating the source-only CNN baseline, its architecture and main training hyperparameters were re-optimized using only source-domain training and validation data. The optimized CNN consists of three convolutional layers with 32, 64, and 128 output channels and kernel sizes of 5, 3, and 3, respectively, followed by global average pooling and a fully connected classifier. The learning rate, dropout rate, batch size, and number of training epochs were set to 1 × 10^−3^, 0.3, 8, and 50, respectively. No target-domain labels or target-domain test performance were used for model selection. For fair comparison, the CNN baseline uses the same 130-dimensional input representation, source/target task definitions, preprocessing and normalization procedure, and repeated-evaluation protocol as the other compared methods.

To improve the statistical reliability of the evaluation, each cross-domain task is independently trained 10 times using random initial parameters, while keeping the target-domain data split fixed. For each run, fault identification accuracy is calculated exclusively on the held-out target test set, and the mean accuracy over the 10 runs is reported in Table 8. The variability across the 10 independent runs was also monitored to assess the stability of repeated training. It can be observed that the proposed method consistently achieves high identification accuracy across all cross-domain tasks. Across the 12 cross-domain tasks, the proposed FE-MK-DANN achieves an average identification accuracy of 92.9%, compared with 69.7%, 87.9%, and 83.4% for the optimized CNN, TCNN, and DANN models, respectively. The proposed method therefore improves the average accuracy by 23.2, 5.0, and 9.5 percentage points over the three comparison methods. Although systematic optimization substantially improves the source-only CNN baseline, a clear performance degradation remains under cross-specimen transfer. Compared with DANN and TCNN, the proposed method also maintains higher average accuracy, indicating the benefit of combining feature enhancement with multi-kernel domain adaptation.

**Figure A1:**
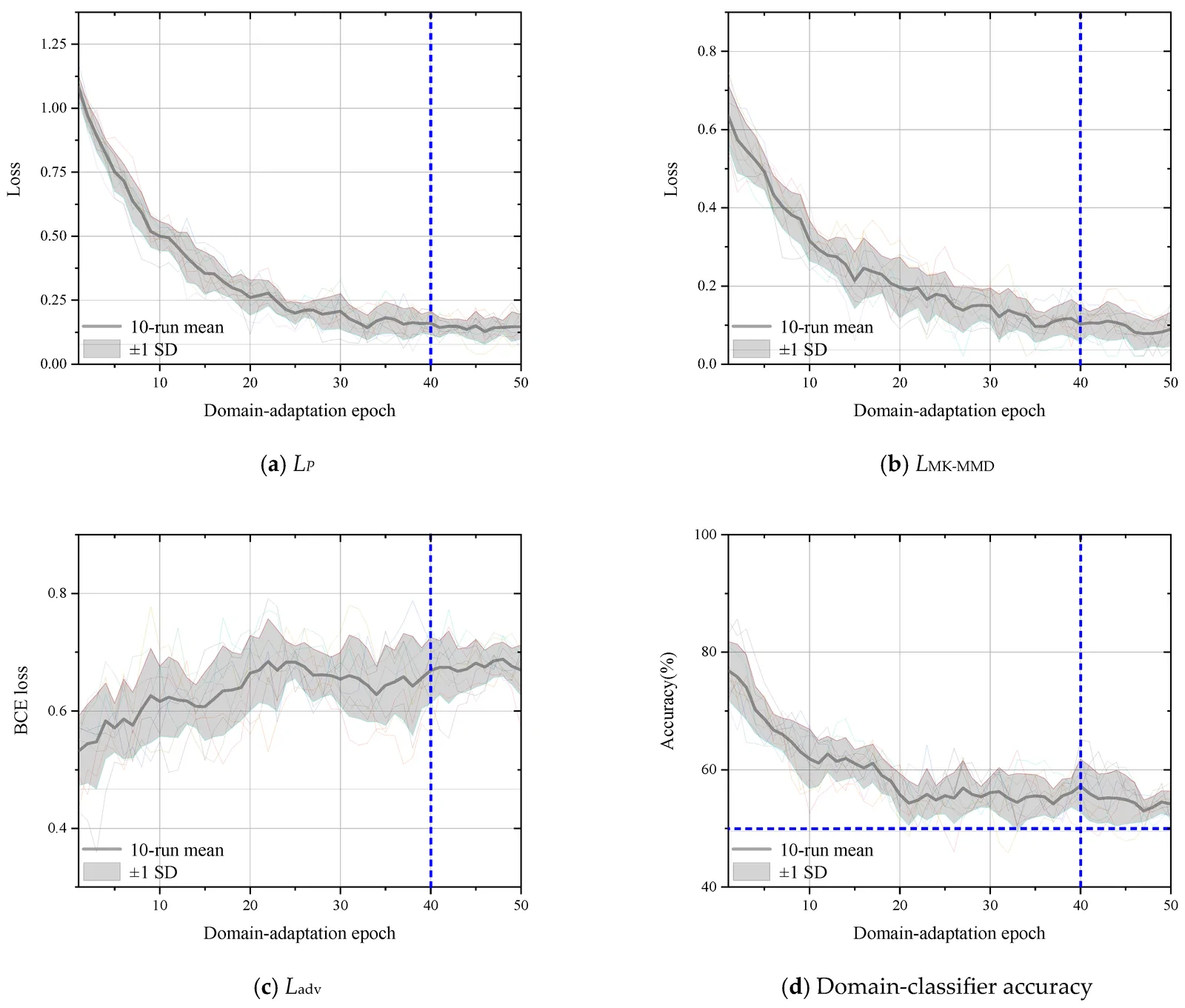
The training curves of *T_A_*_1_.

**Figure A2:**
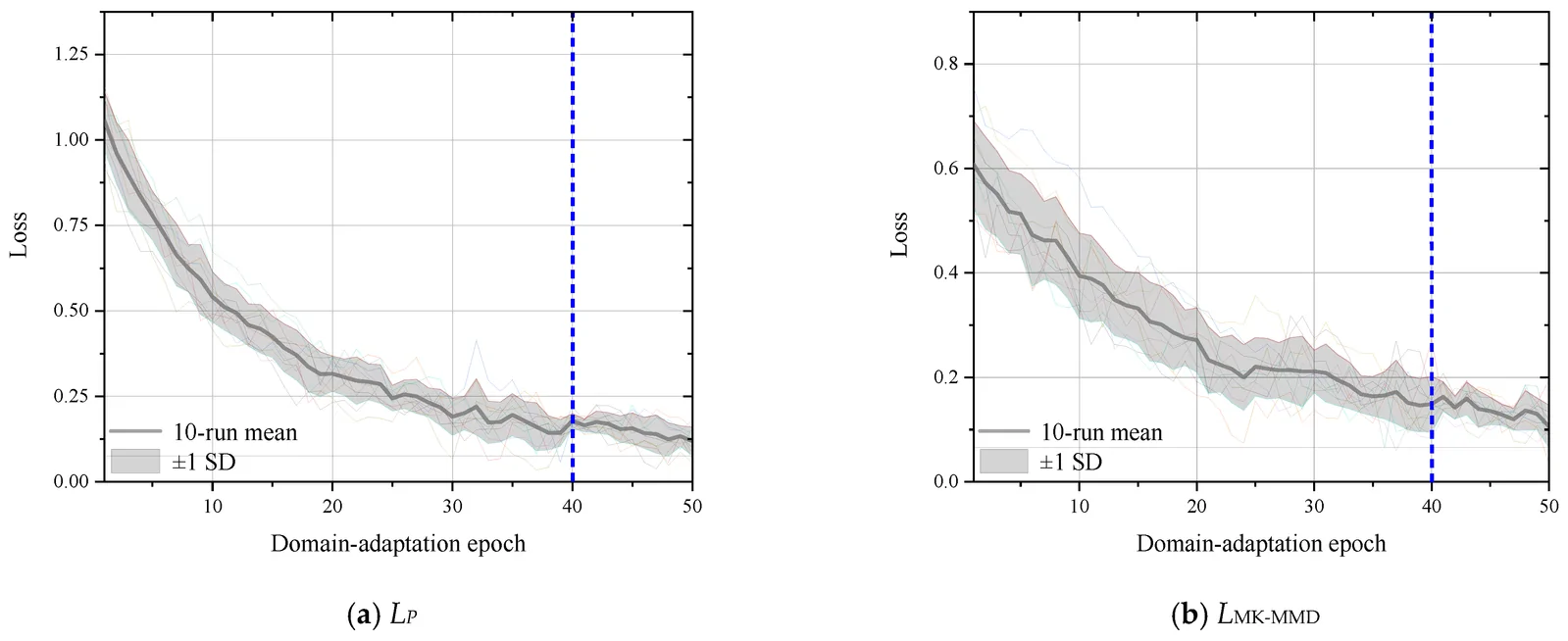

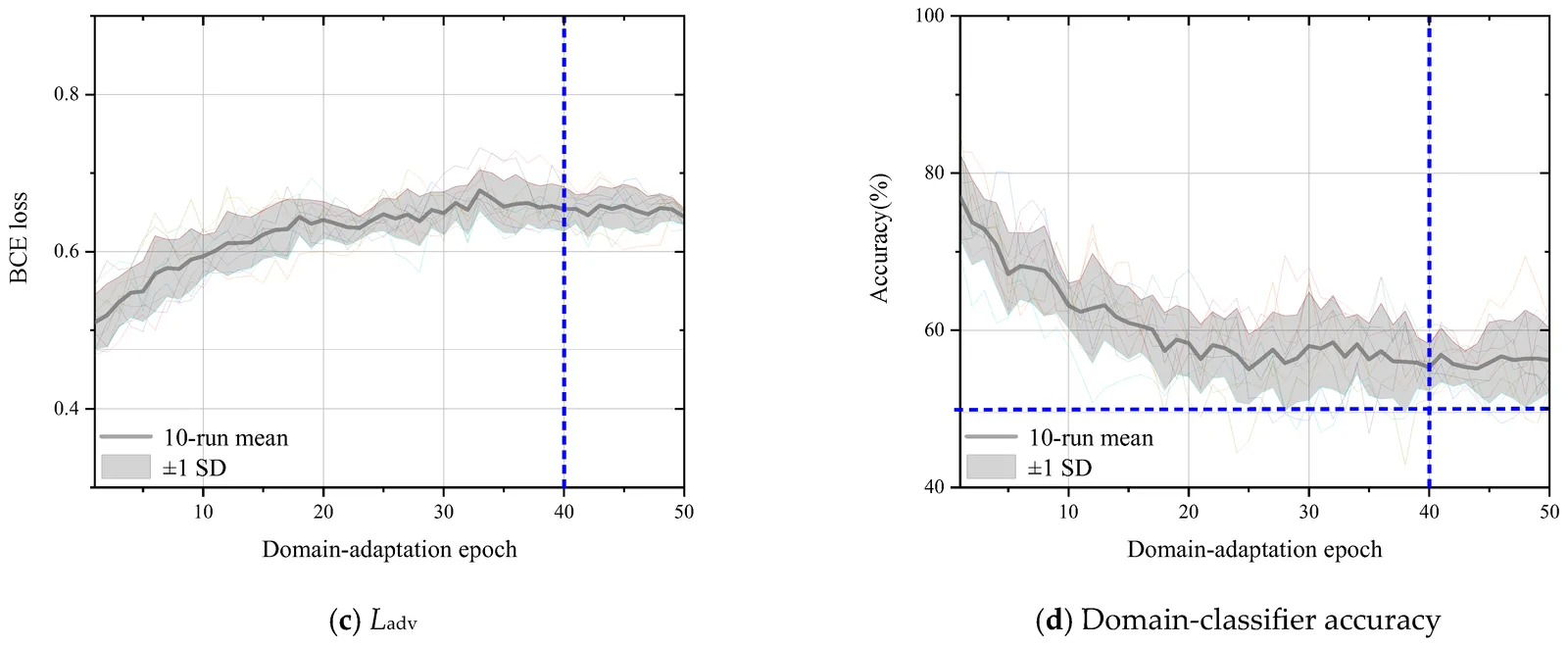
The training curves of *T_A_*_2_.

**Figure A3:**
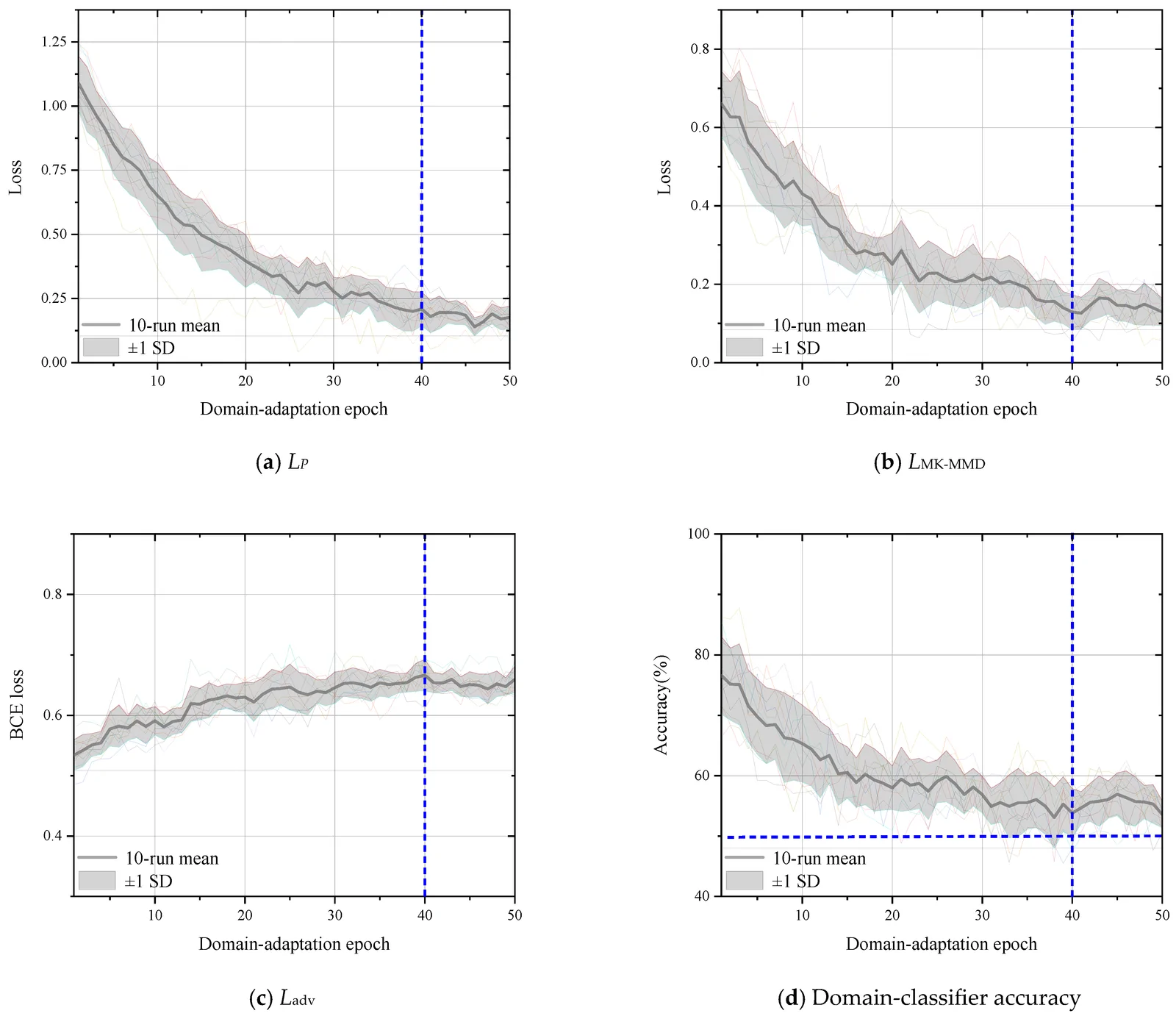
The training curves of *T_A_*_3_.

**Figure A4:**
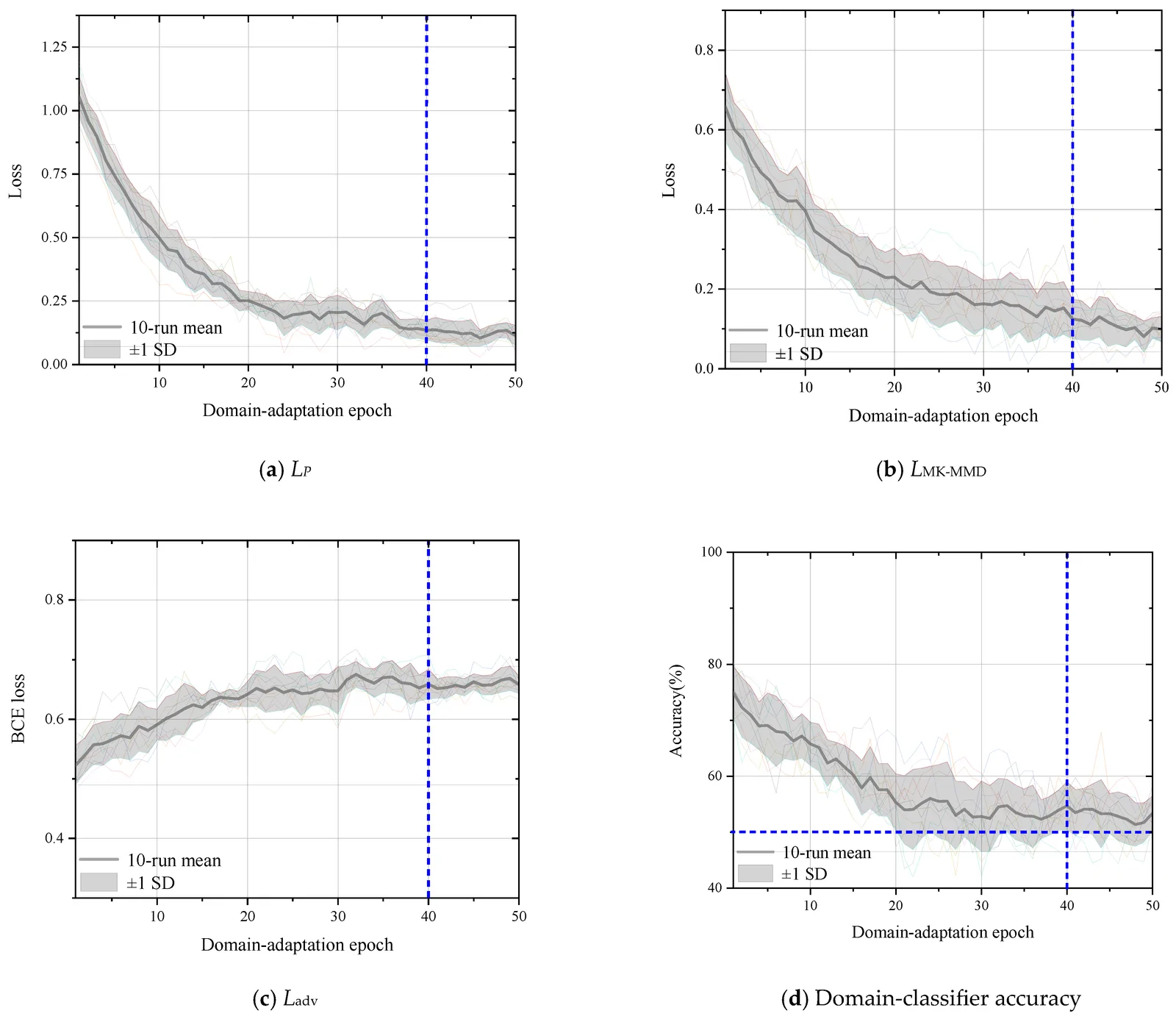
The training curves of *T_A_*_4_.

**Figure A5:**
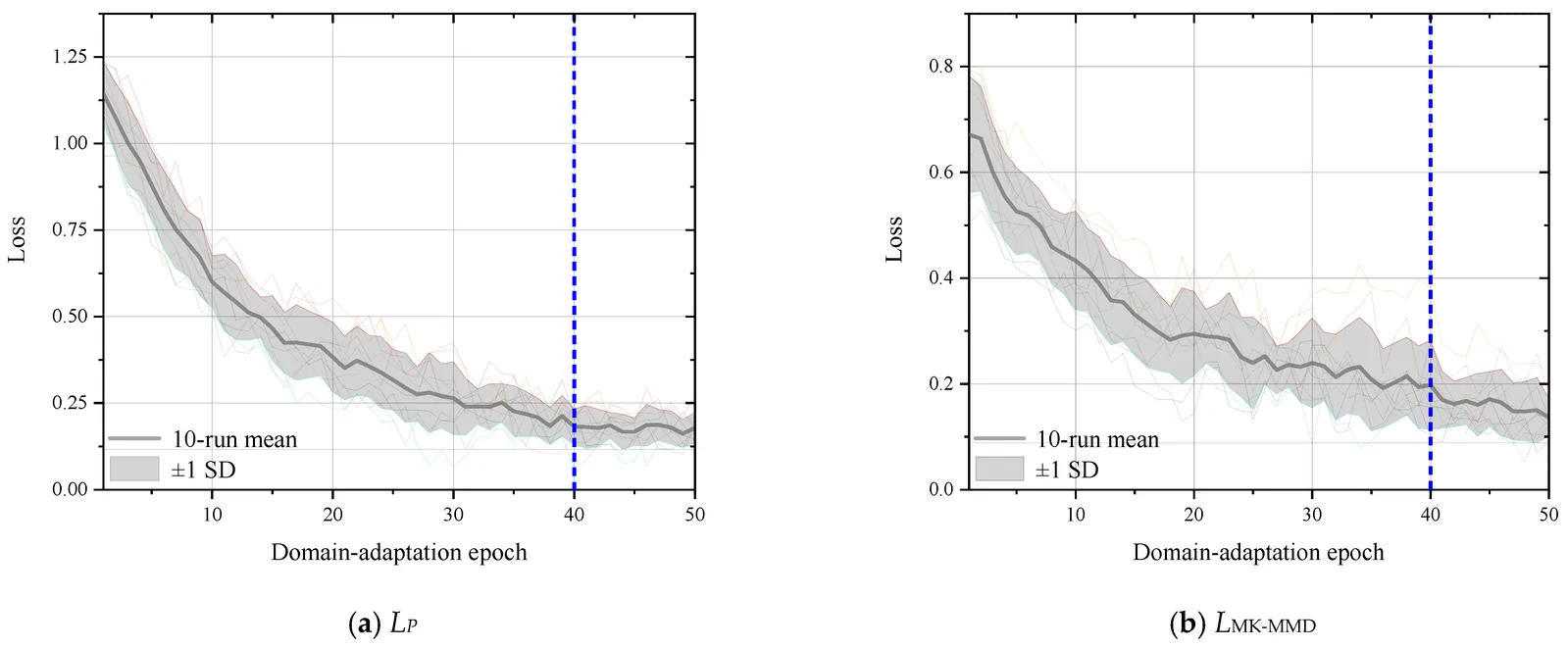

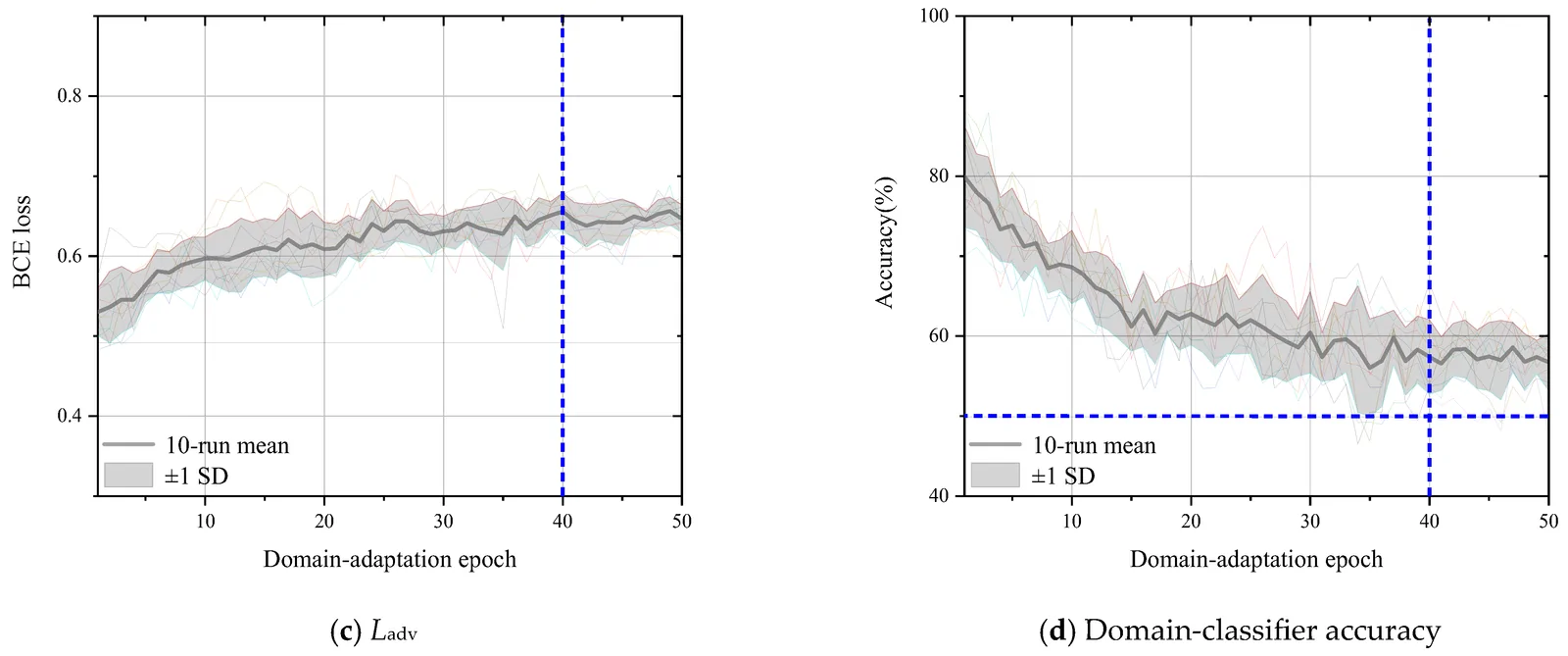
The training curves of *T_A_*_5_.

**Figure A6:**
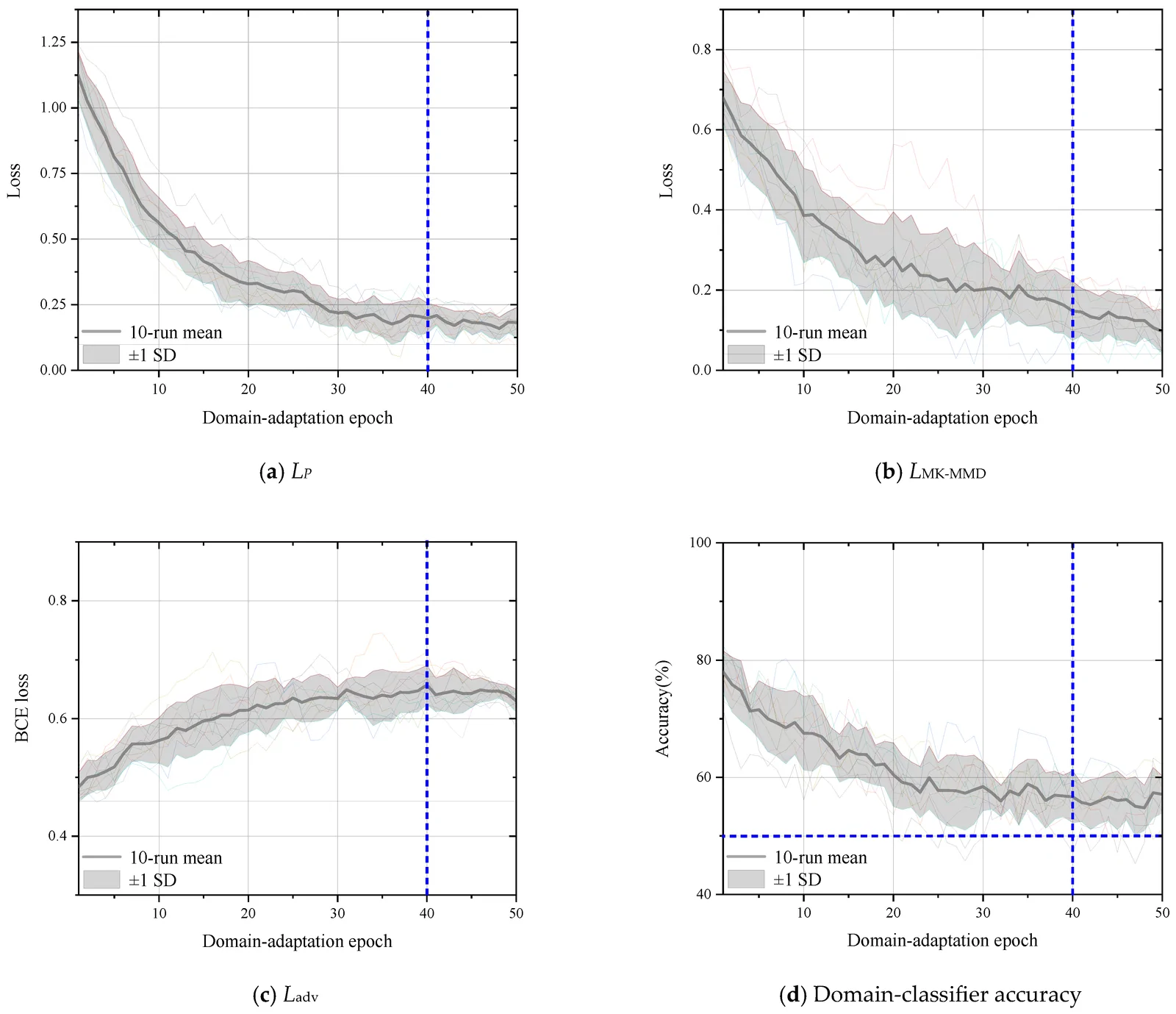
The training curves of *T_A_*_6_.

**Figure A7:**
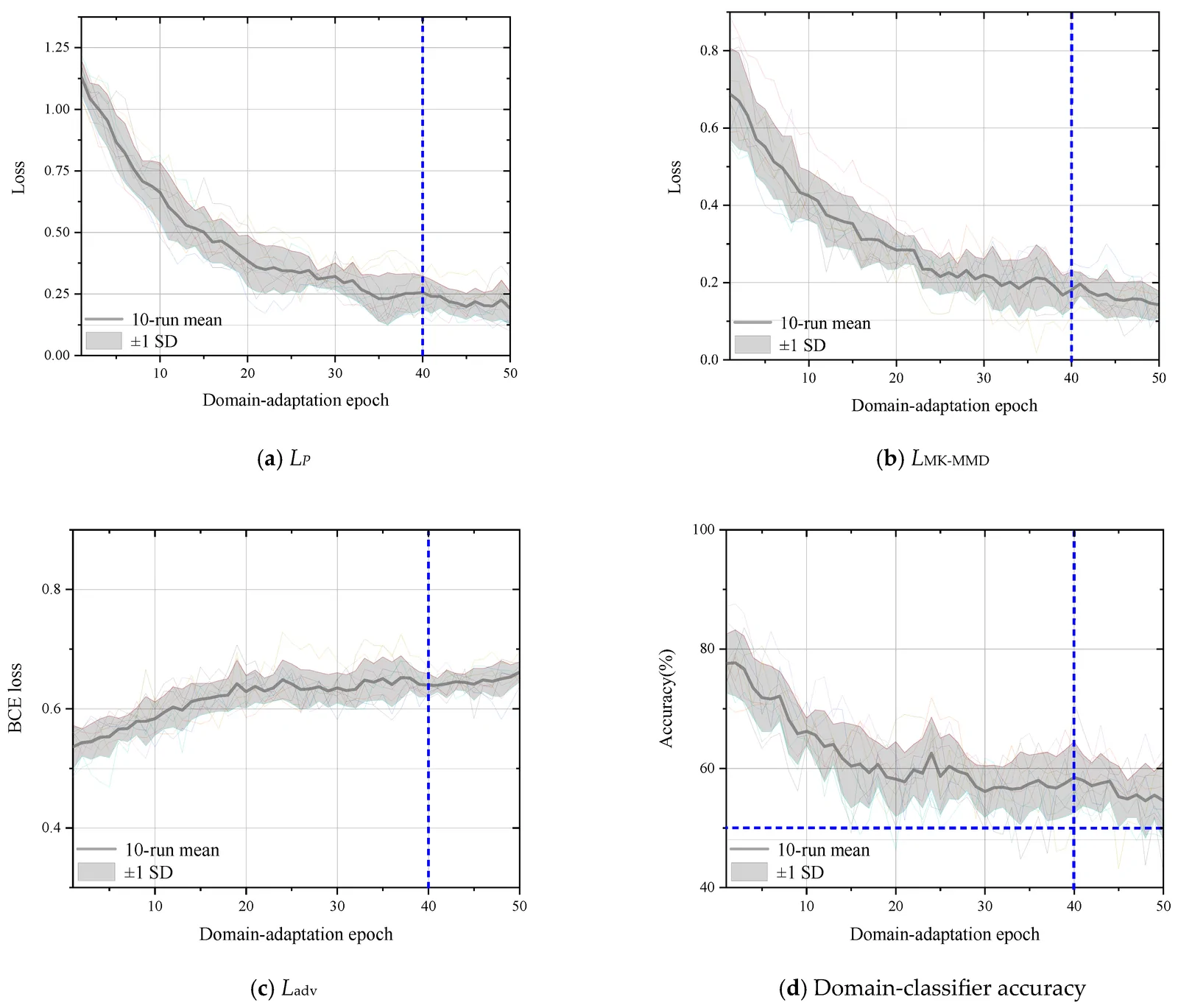
The training curves of *T_B_*_1_.

**Figure A8:**
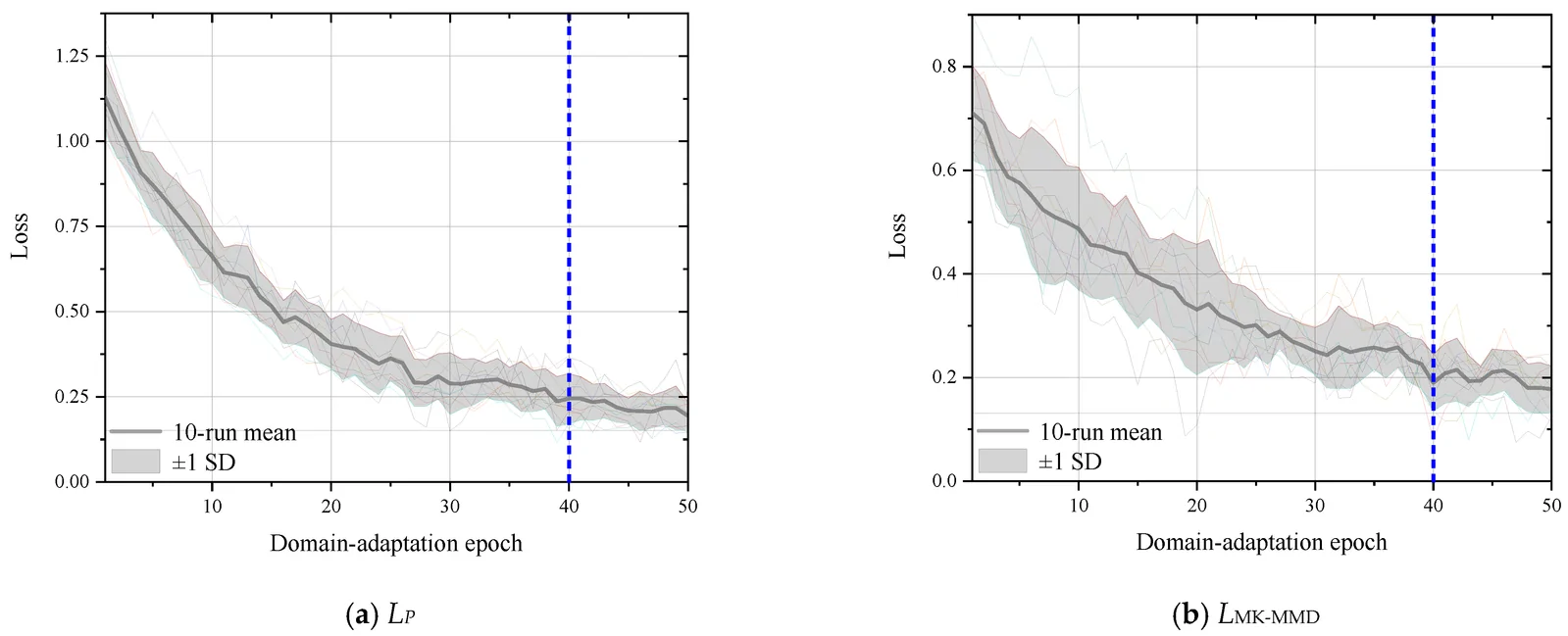

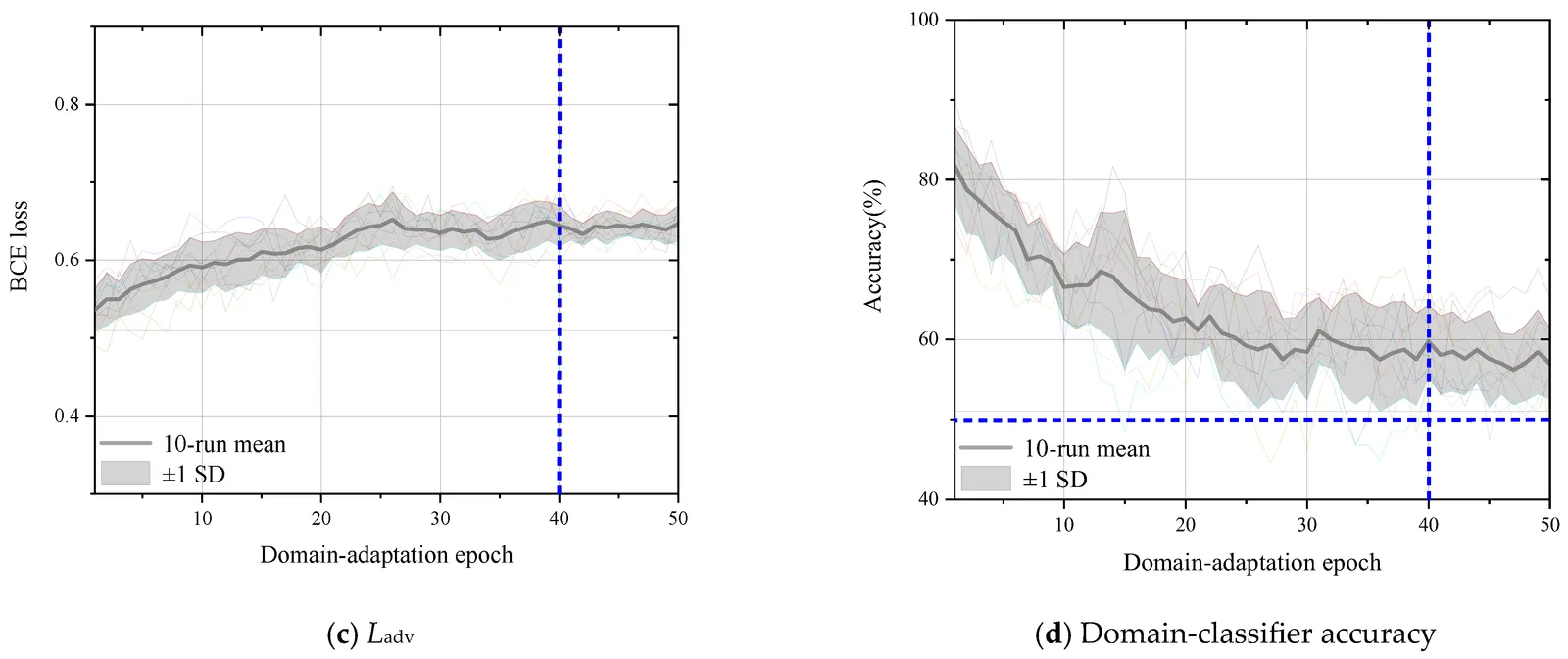
The training curves of *T_B_*_2_.

**Figure A9:**
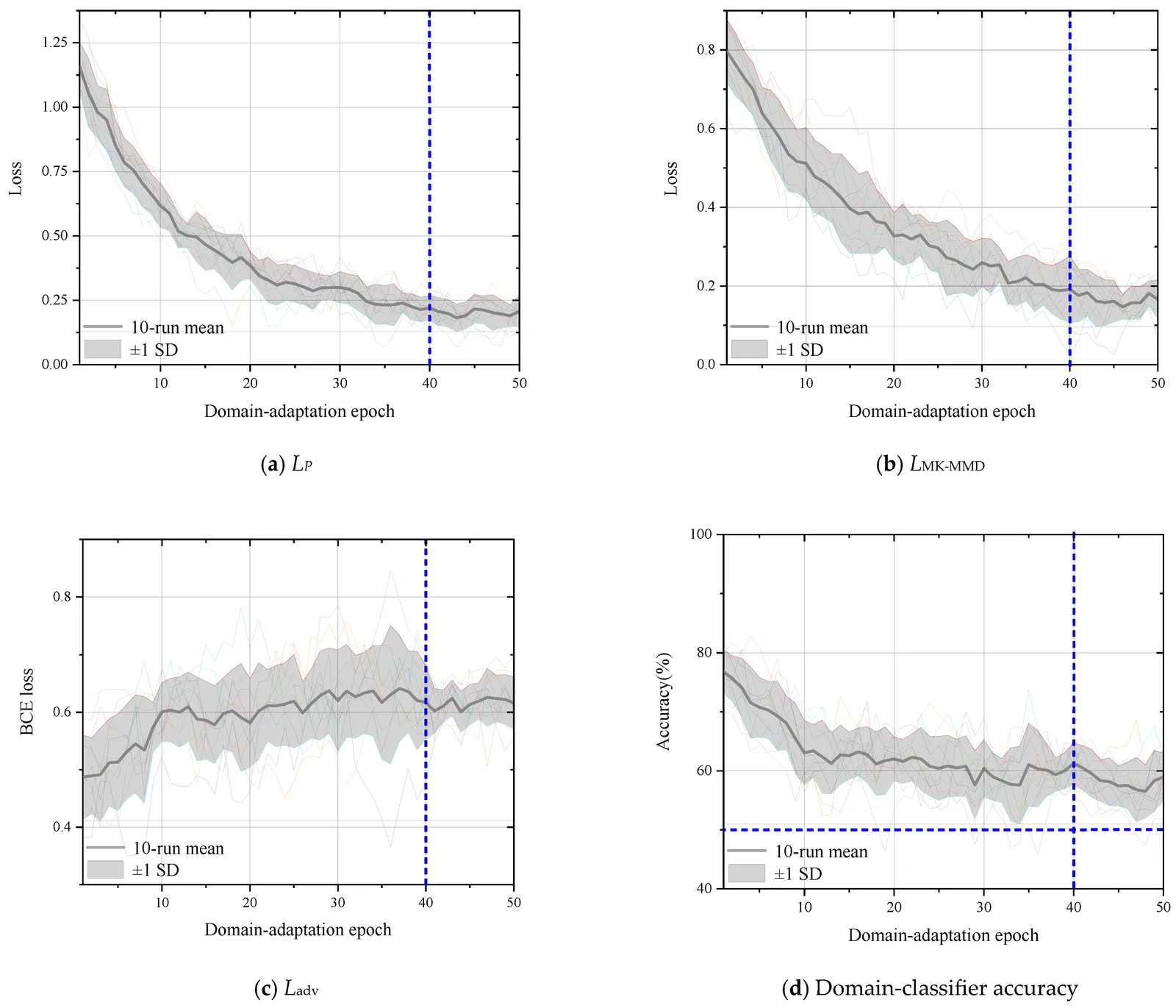
The training curves of *T_B_*_3_.

**Figure A10:**
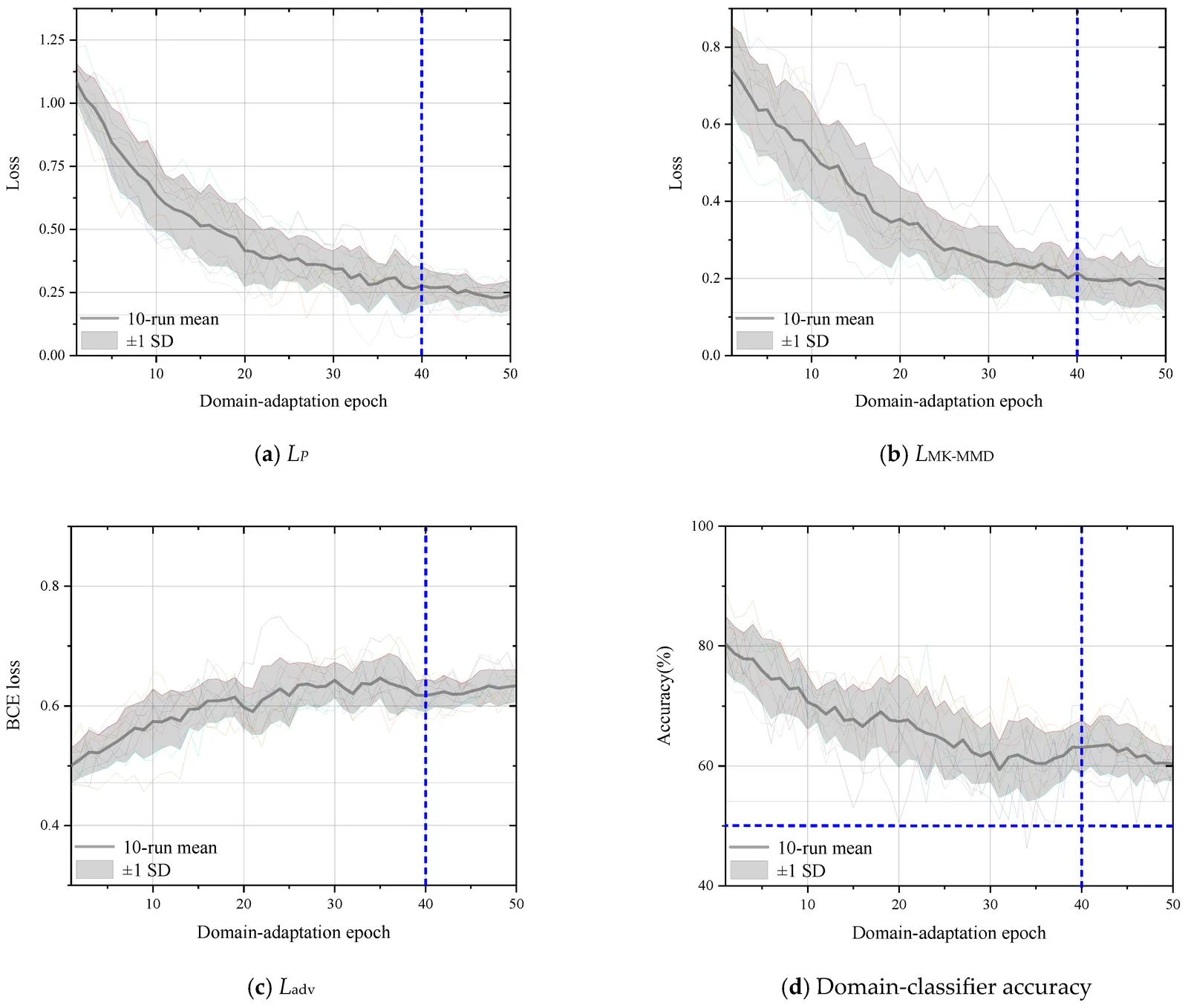
The training curves of *T_B_*_4_.

**Figure A11:**
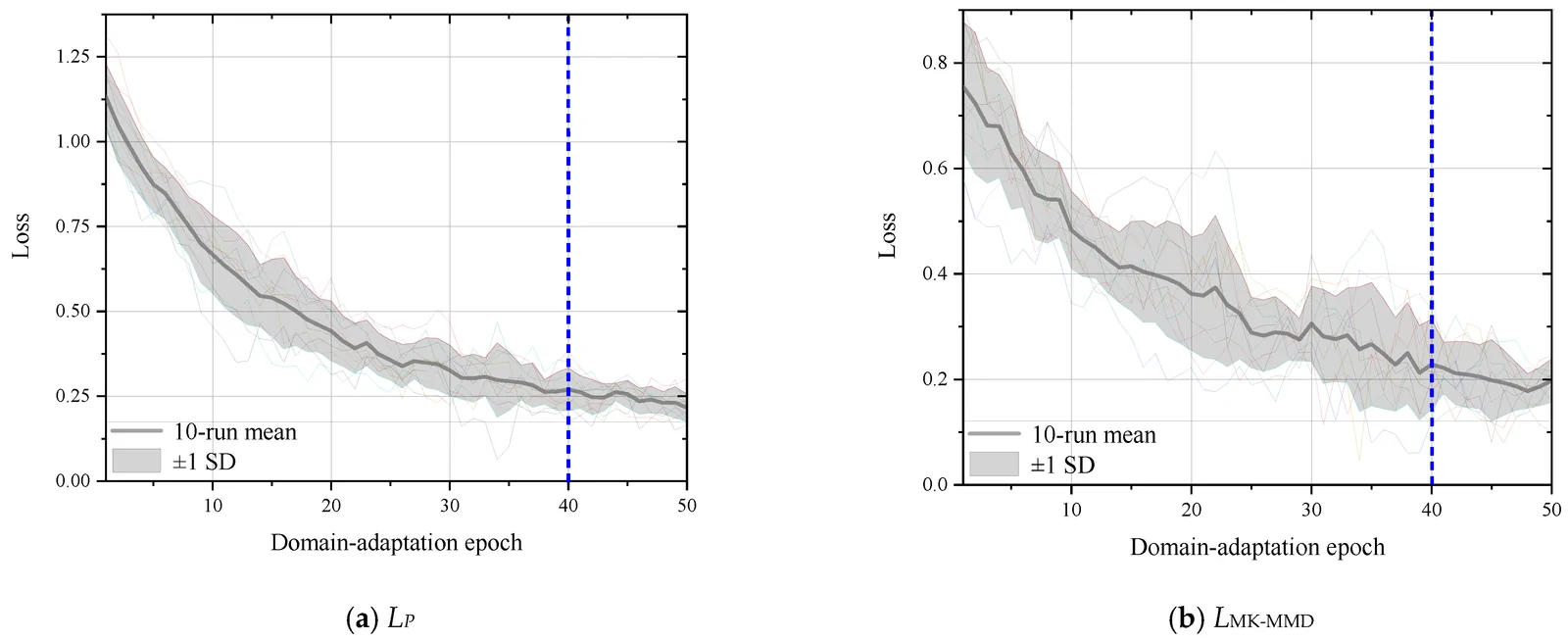

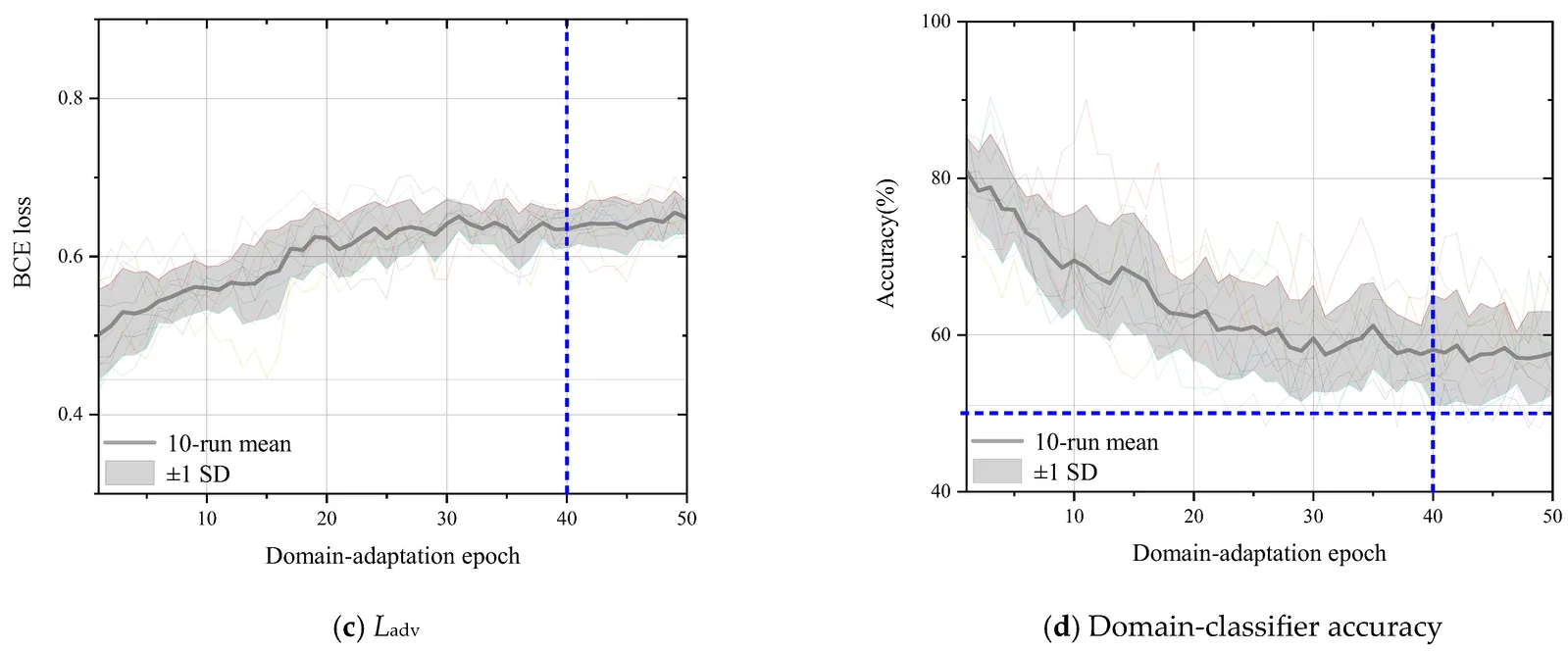
The training curves of *T_B_*_5_.

**Figure A12:**
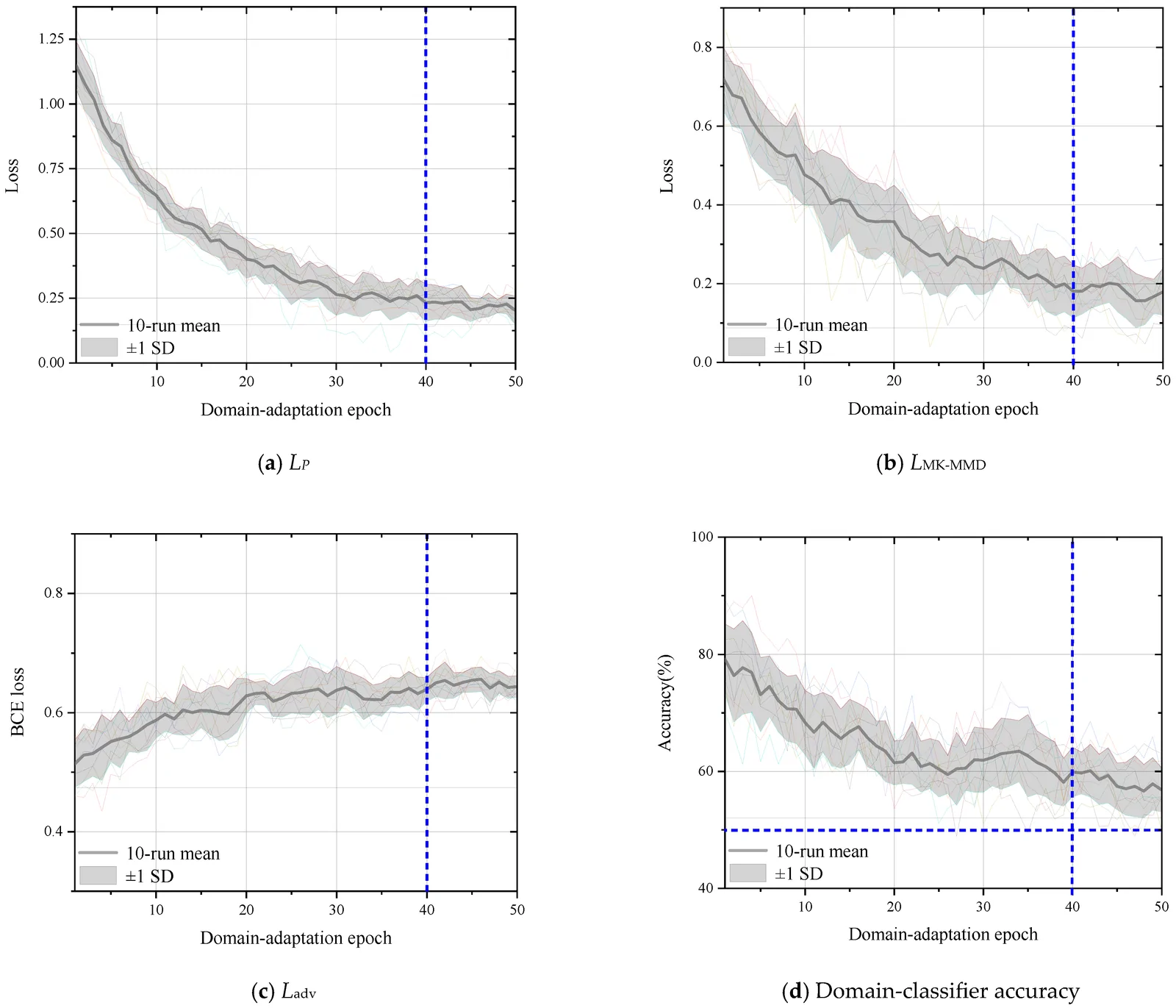
The training curves of *T_B_*_6_.

To further examine the convergence behavior of the adversarial adaptation, the training trajectories of *L_P_*, *L*_MK-MMD_, *L*_adv_, and the domain-classifier accuracy for all 12 tasks are provided in Appendix B (Figure A1, Figure A2, Figure A3, Figure A4, Figure A5, Figure A6, Figure A7, Figure A8, Figure A9, Figure A10, Figure A11 and Figure A12). Overall, *L_P_* and *L*_MK-MMD_ progressively decrease and stabilize, while *L*_adv_ gradually approaches the regime associated with weakened domain discriminability. Meanwhile, the domain-classifier accuracy decreases substantially from its initial level and remains close to the chance-level region, generally around 50–60% in the later training stage. These results indicate that the source and target representations become increasingly difficult to distinguish, suggesting that the adversarial training reaches an effective domain-confusion state without evident premature convergence or training failure.

To further assess the influence of spatial correlation, the proposed method was evaluated using the block-wise spatial splitting strategy described in Section 4.3, as shown in Table 9. The average accuracy decreases from 92.9% to 90.8% under block-wise spatial splitting, corresponding to a reduction of approximately 2.1 percentage points. The task-specific reductions range from 0.9 to 3.5 percentage points. These results indicate that spatial correlation introduces a moderately optimistic bias under random splitting, while the proposed method still maintains comparable cross-domain performance under the stricter block-wise evaluation.

**Figure 10:**
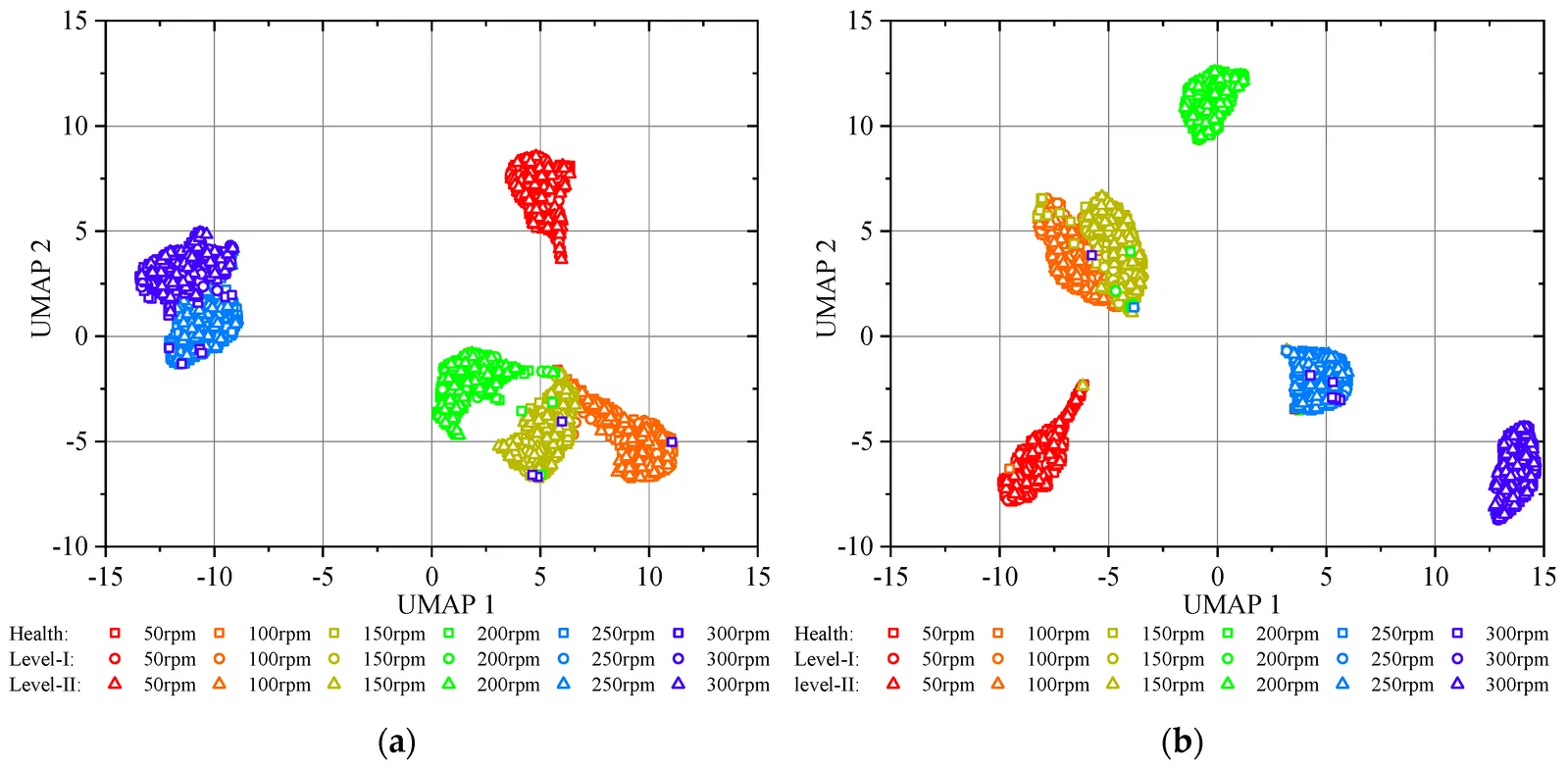
Initial feature distributions of ball screws under various operating conditions: (**a**) Specimen 1; (**b**) Specimen 2.

To investigate the feature distribution characteristics under different operating conditions, UMAP is utilized to project the raw vibration features into a low-dimensional space. As shown in Figure 10, the samples collected from specimen 1 and specimen 2 exhibit distinct distribution patterns with respect to rotational speed variations, while the samples corresponding to different fault categories remain highly overlapped. This result indicates that the raw vibration feature representation contains substantial operating-condition-related variations, whereas the feature differences associated with fault categories are less pronounced. Consequently, directly using the raw features may lead to limited generalization capability when the model is applied to target operating condition.

**Figure 11:**
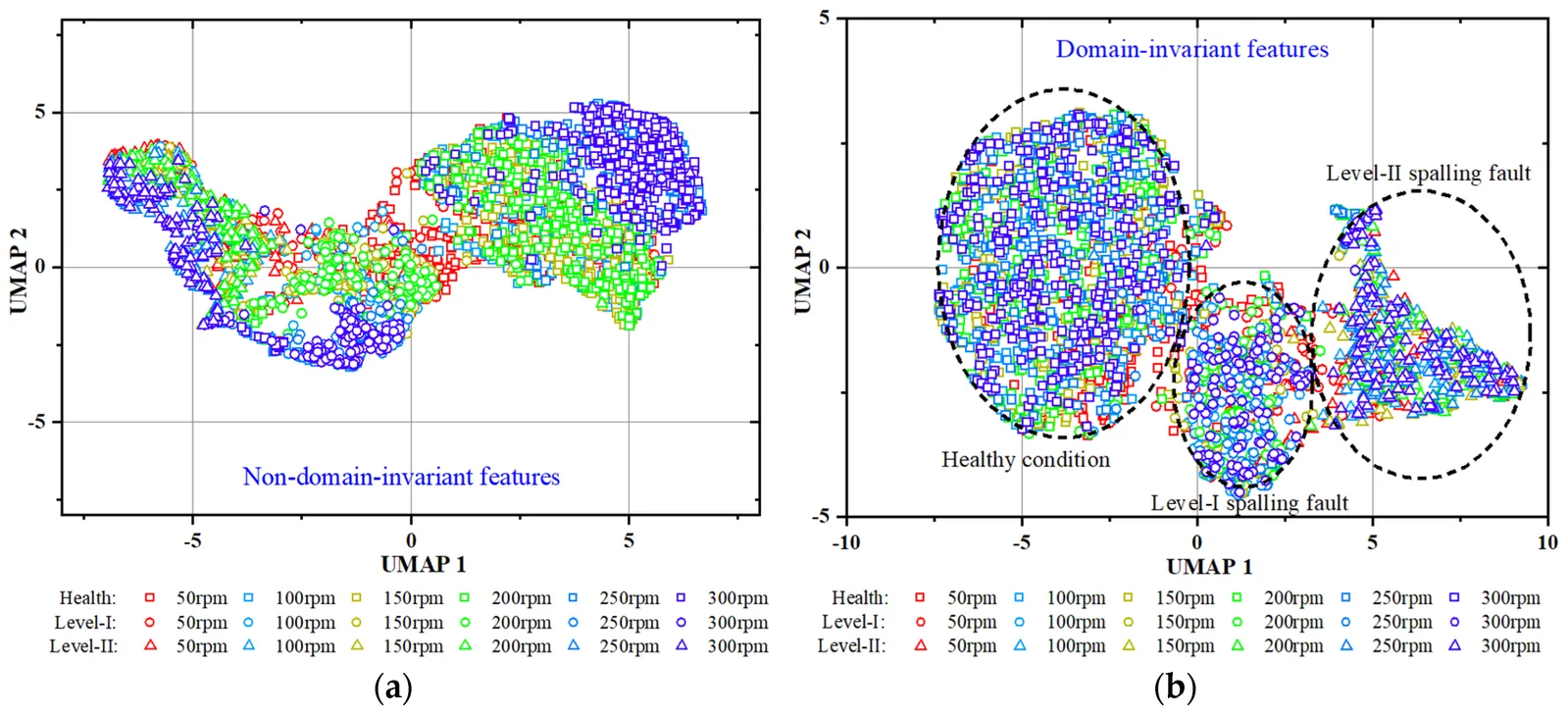
Feature distribution comparison under task $T_{A6}$: (**a**) before domain adaptation; (**b**) after domain adaptation.

To further verify the effectiveness of cross-domain adaptation, Figure 11 compares the feature distributions obtained before and after transfer learning under task $T_{A6}$. Without domain adaptation, as shown in Figure 11a, the feature distributions extracted by the fusion module exhibit distinct clustering patterns according to rotational speed rather than fault category. This observation indicates that the learned representations contain substantial domain-specific information, while common fault-discriminative information across domains is insufficiently captured. Consequently, the model may suffer from performance degradation when generalized to target operating conditions. After adversarial domain adaptation and MK-MMD-based distribution alignment, as shown in Figure 11b, the feature distributions corresponding to different domains become substantially aligned. Specifically, samples with identical fault states but different rotational speeds are projected into closer regions of the feature space, indicating that the proposed FE-MK-DANN effectively reduces domain-specific variations while preserving fault-discriminative information. These results demonstrate that the proposed multi-kernel adversarial domain adaptation strategy improves the robustness and generalization capability of fault identification under varying operating conditions.

**Figure 12:**
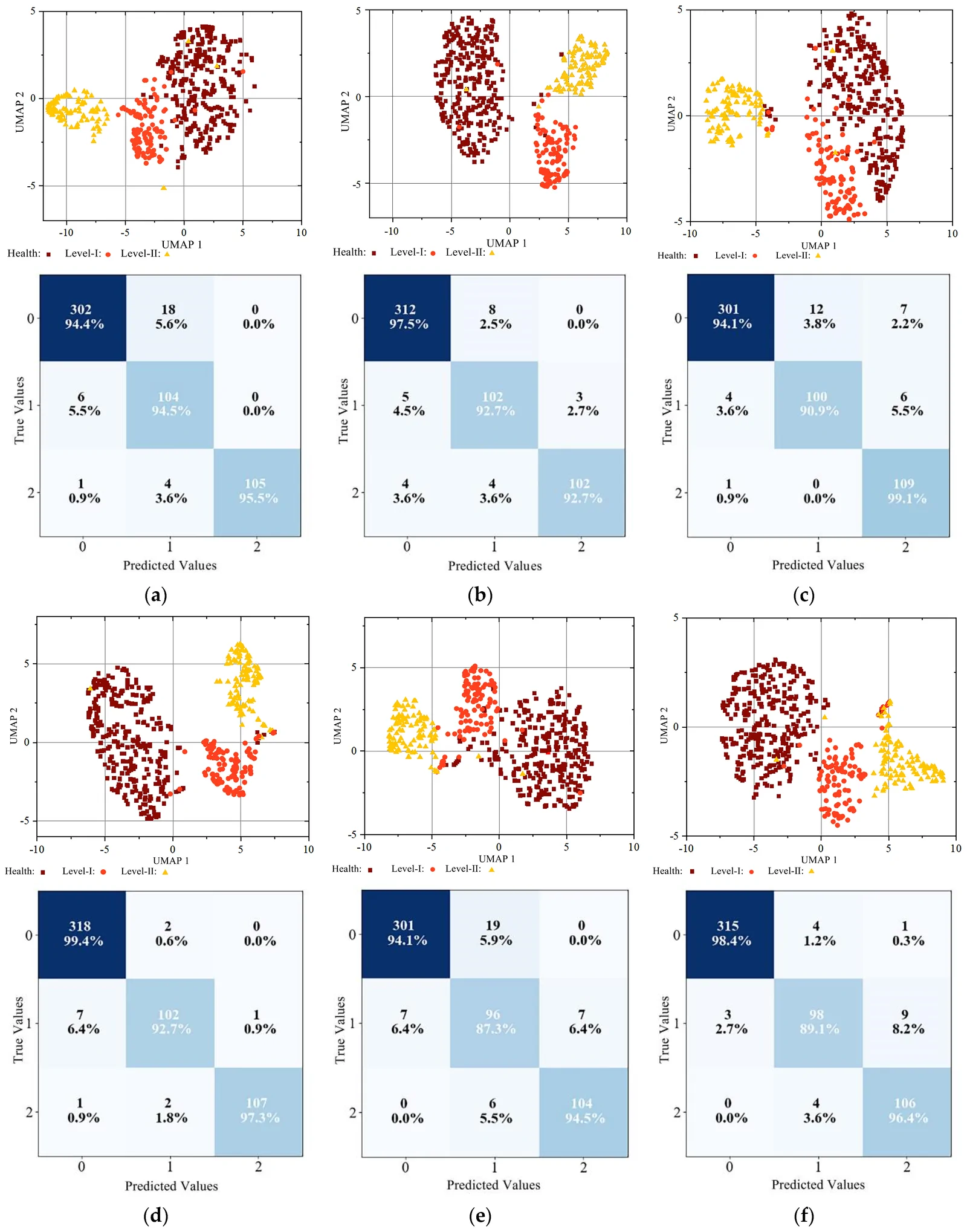
UMAP feature distributions and confusion matrices for the six intra-specimen cross-speed tasks: (**a**) $T_{A1}$; (**b**) $T_{A2}$; (**c**) $T_{A3}$; (**d**) $T_{A4}$; (**e**) $T_{A5}$; (**f**) $T_{A6}$.

**Figure 13:**
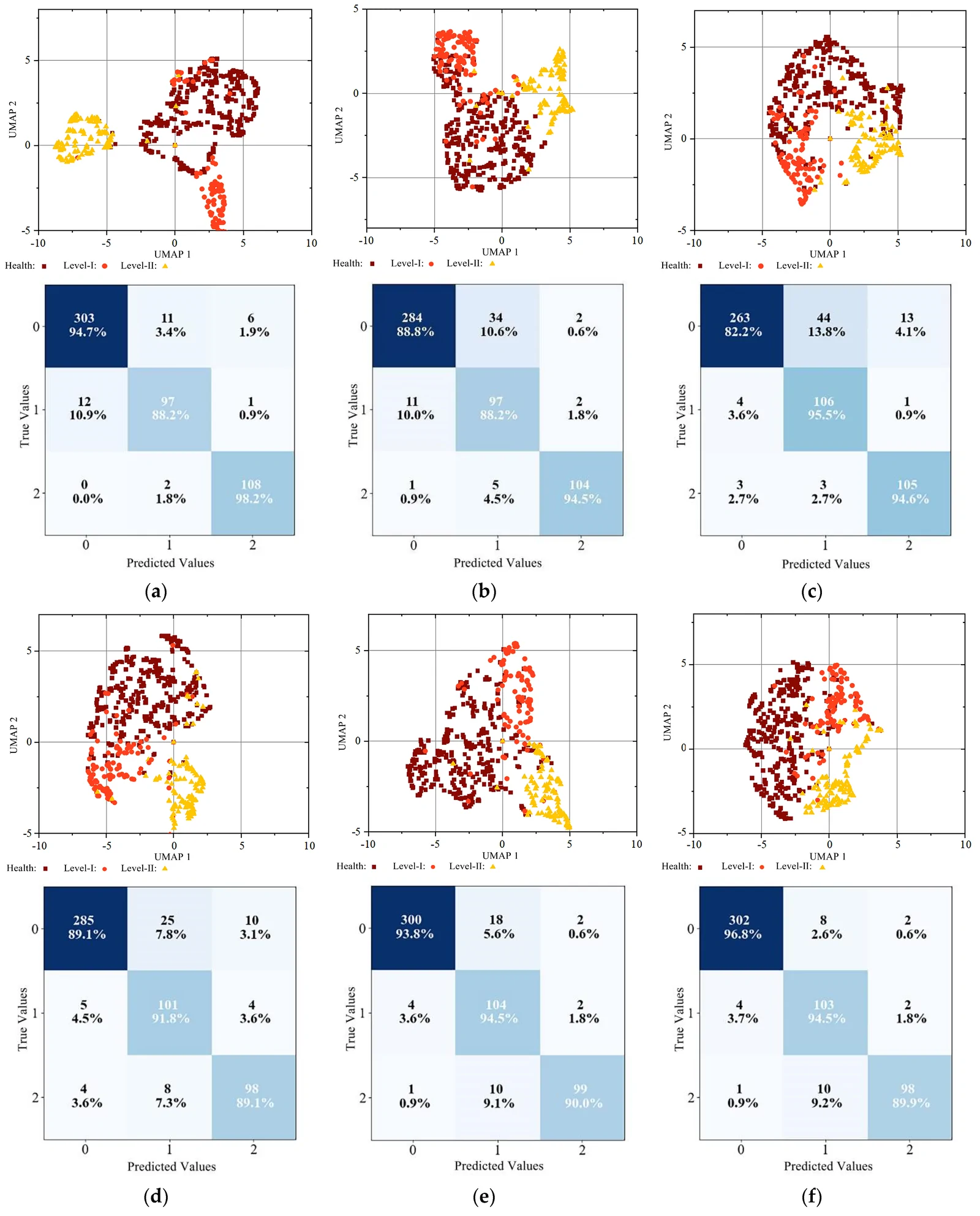
UMAP feature distributions and confusion matrices for the six cross-specimen tasks: (**a**) $T_{B1}$; (**b**) $T_{B2}$; (**c**) $T_{B3}$; (**d**) $T_{B4}$; (**e**) $T_{B5}$; (**f**) $T_{B6}$.

The detailed identification results of different cross-domain tasks are presented in Figure 12 and Figure 13. The figures include the UMAP-based visualization of feature distributions with different fault labels and the corresponding confusion matrices, providing a comprehensive evaluation of the fault identification performance of the proposed model. As shown in Figure 12, for the cross-domain tasks $T_{A1}$–$T_{A6}$, the features extracted by the feature fusion module exhibit strong clustering characteristics for level-II spalling faults, with relatively clear decision boundaries between this class and the other categories. This indicates that level-II spalling faults possess higher feature separability and are easier for the model to distinguish. However, in tasks $T_{A1}$ and $T_{A3}$, the feature distributions of level-I spalling faults and healthy states exhibit partial overlap, suggesting relatively poor discriminability of these fault features. Furthermore, the boundaries among different feature clusters become clearer in several tasks at higher rotational speeds. This observation indicates that the measured vibration features exhibit improved empirical separability under these operating conditions. However, this observation is interpreted only from the feature-distribution perspective and does not imply a specific physical excitation mechanism. In contrast, as shown in Figure 13, for the cross-domain tasks $T_{B1}$–$T_{B6}$, the feature distributions of different fault categories maintain relatively compact clusters, but the corresponding decision boundaries remain less distinct. This result reflects the increased difficulty of fault identification under cross-specimen transfer scenarios. The manufacturing-induced geometric deviations and assembly discrepancies between different ball screws may introduce specimen-dependent variations in the measured vibration responses, causing the same fault type to exhibit different feature representations across specimens. According to the results in Table 8, the optimized source-only CNN exhibits a clear reduction in identification performance under the cross-specimen tasks $T_{B1}$–$T_{B6}$, with its average accuracy decreasing from 78.6% under cross-speed transfer to 60.8% under cross-specimen transfer. This indicates that specimen-dependent distribution shifts remain challenging without explicit domain adaptation. In contrast, the proposed FE-MK-DANN maintains consistently higher identification performance across the cross-specimen transfer tasks, indicating the benefit of explicit domain alignment in mitigating specimen-dependent feature-distribution differences.

### 4.5. Ablation Study

To evaluate the contributions of the main components of FE-MK-DANN, ablation experiments are conducted on *T_A4_*, *T_B3_*, and *T_B5_*. All variants use the same input representation, data partitions, preprocessing procedure, and evaluation protocol as the complete model, with only the investigated component or training strategy modified. The results are summarized in Table 10.

The complete FE-MK-DANN achieves the highest average accuracy of 91.7% across the three tasks. Removing the attention module or replacing the residual feature-fusion structure with a plain convolutional trunk reduces the average accuracy to 89.7% and 90.1%, respectively. Replacing MK-MMD with single-kernel MMD or using adversarial adaptation/MK-MMD alone further degrades performance, while end-to-end joint training achieves 89.8%. These results quantitatively demonstrate that the attention module, residual feature-fusion structure, complementary adversarial/MK-MMD alignment, and two-stage training strategy each contribute to the final performance.

In addition to the improvement in identification accuracy, the proposed framework offers complementary advantages in feature representation and domain alignment. The attention mechanism suppresses redundant and noise-related components, while the residual feature-fusion structure facilitates hierarchical feature learning and stable information propagation. Moreover, adversarial learning and MK-MMD provide complementary domain-alignment mechanisms without requiring target-domain fault labels. Nevertheless, the proposed method follows a transductive UDA setting and therefore requires unlabeled measurements from each new target domain and an additional adaptation stage before inference, which increases the data-collection and computational requirements during deployment.

## 5. Conclusions

This study investigates the cross-domain fault identification problem of ball screw feed systems under variable operating conditions. A feature-enhanced multi-kernel domain adaptation framework is proposed to address the distribution discrepancies caused by variations in rotational speed and ball screw specimens. The proposed framework learns transferable fault representations directly from experimentally measured vibration features without introducing dynamic models or physics-based prior information. The main conclusions are summarized as follows.

- The proposed FE-MK-DANN framework demonstrates robust fault identification performance across the 12 investigated cross-domain tasks. The proposed method achieves an average identification accuracy of 92.9%, compared with 69.7%, 87.9%, and 83.4% for the optimized CNN, TCNN, and DANN models, respectively. Although systematic optimization substantially improves the source-only CNN baseline, its average accuracy decreases from 78.6% under cross-speed transfer to 60.8% under cross-specimen transfer, indicating that specimen-dependent distribution shifts remain challenging without explicit domain adaptation.
- Ablation experiments on *T_A4_*, *T_B3_*, and *T_B5_* show that both the attention-based feature enhancement module and the residual feature-fusion structure contribute to cross-domain fault identification performance. Removing the attention module decreases the average accuracy across the three tasks by 2.0 percentage points, while replacing the residual feature-fusion structure with a plain convolutional trunk reduces the average accuracy by 1.6 percentage points. These results provide direct experimental evidence for the contributions of the two architectural components.
- Ablation experiments further show that combining adversarial adaptation with MK-MMD provides additional benefits for cross-domain fault identification compared with using either mechanism alone. The complete model achieves an average accuracy of 91.7% on the three ablation tasks, compared with 87.7% and 87.6% for adversarial-only and MK-MMD-only adaptation, respectively. Replacing MK-MMD with single-kernel MMD also reduces the average accuracy to 88.8%, while end-to-end joint training achieves 89.8%, supporting the use of multi-kernel distribution alignment and the proposed two-stage training strategy.

The proposed framework follows a transductive UDA setting and requires unlabeled target-domain samples for adaptation before inference, introducing additional data-acquisition and computational costs for a new ball screw. The current validation is limited to two specimens, local spalling faults, and six rotational speeds from 50 to 300 rpm. Its effectiveness for other fault types remains to be experimentally verified. Future work will extend the validation scope and investigate domain generalization and source-free/online adaptation to reduce dependence on target-domain data.

## Figures and Tables

**Table 1:** Comparison with related cross-domain fault diagnosis methods.

Method	Feature Enhancement/Preprocessing	Residual Feature Extraction	Adversarial Alignment	Distribution Alignment	Transfer Scenario
Azamfar et al. [25]	-	-	-	MMD	Cross-condition
Pandhare et al. [26]	-	-	-	MMD	Cross-sensor/location
Xie et al. [29]	Wavelet convolution	-	-	JDA	Cross-sensor/location
Zhou et al. [27]	ICEEMDAN-MICOA-VMD + SDP	SE-Resformer	√	-	Cross-condition
Proposed FE-MK-DANN	Attention-based feature enhancement	√	√	MK-MMD	Cross-speed & cross-specimen

**Table 2:** Specifications of the feed drive test rig.

Parameters	Values	Parameters	Values
Ball radius of the linear guideway (mm)	3.175	Poisson’s ratio	0.3
Raceway radius of the rail (mm)	3.3	Elastic modulus (GPa)	207
Initial contact angle (°)	45	Mass of the worktable (kg)	170
Number of balls per row in the linear guideway	12	Ball radius of the bearing (mm)	4.4
Preload of the linear guideway (N)	4830	Raceway radius of the bearing (mm)	4.6
Carriage width (mm)	70	Number of balls in the bearing	13
Rail width (mm)	35	Contact angle of the bearing (°)	40

**Table 3:** Parameters of the 4010-ball screw.

Parameters	Values	Parameters	Values
Ball radius (mm)	2.9765	Nominal diameter of the screw (mm)	40
Ball mass (kg)	0.0008	Raceway conformity ratio	0.555
Contact angle of the ball screw (°)	45	Lead of the screw (mm)	10
Δs length (mm)	11	γ angle (°)	40
Number of balls in the nuts	126	Nut mass (kg)	1
Total number of return tubes	6	Included angle between adjacent deflectors (°)	120
Spacer stiffness (N/m)	6.79 × 10^9^	Screw shaft mass (kg)	16
Screw length (mm)	1570	Young’s modulus (GPa)	206

**Table 4:** Configuration of the experimental vibration dataset.

Specimen No.	Operating Condition	Fault Condition	Feature Dimension	Number of Samples	Signal Type
1	50 rpm	Healthy condition	16 time-domain features	12 × 540	Vibration signal
	100 rpm				
	150 rpm				
	200 rpm				
	250 rpm	Level-I spalling fault	14 frequency-domain features		
	300 rpm				
2	50 rpm				
	100 rpm				
	150 rpm	Level-II spalling fault	100 frequency-bin components		
	200 rpm				
	250 rpm				
	300 rpm				

**Table 5:** Cross-domain task configurations for measured fault data.

Task ID	Description	Task ID	Description
$T_{A1}$	Intra-specimen identification at 50 rpm	$T_{B1}$	Cross-specimen identification at 50 rpm
$T_{A2}$	Intra-specimen identification at 100 rpm	$T_{B2}$	Cross-specimen identification at 100 rpm
$T_{A3}$	Intra-specimen identification at 150 rpm	$T_{B3}$	Cross-specimen identification at 150 rpm
$T_{A4}$	Intra-specimen identification at 200 rpm	$T_{B4}$	Cross-specimen identification at 200 rpm
$T_{A5}$	Intra-specimen identification at 250 rpm	$T_{B5}$	Cross-specimen identification at 250 rpm
$T_{A6}$	Intra-specimen identification at 300 rpm	$T_{B6}$	Cross-specimen identification at 300 rpm

**Table 6:** Architecture parameters of the proposed FE-MK-DANN framework.

Component	Layer Name	Symbol	Kernel Size	Output Size
Feature enhancement module $G_{A}$	Input	input	/	8 × 1 × 130
	Self-attention weighting	Conv1	1 × 1 × 3 × 1	8 × 1 × 130
		Self-Attention	3	8 × 1 × 130
		Concat	/	8 × 4 × 130
		Conv2	8 × 4 × 3 × 1	8 × 8 × 130
Feature fusion module $G_{G}$	Residual connection	Conv3	32 × 8 × 3 × 1	8 × 32 × 130
		ReLU	/	8 × 32 × 130
		Conv4	64 × 32 × 3 × 1	8 × 64 × 130
		LayerNorm	64	8 × 64 × 130
		ReLU	/	8 × 64 × 130
		Add	/	8 × 32 × 130
		Conv5	32 × 64 × 3 × 1	8 × 32 × 130
		ReLU	/	8 × 32 × 130
		Conv6	32 × 32 × 3 × 1	8 × 32 × 130
		LayerNorm	32	8 × 32 × 130
		ReLU	/	8 × 32 × 130
		Add	/	8 × 32 × 130
	Feature fusion	Average-Pooling	/	8 × 32
		Linear 1	32 × 32	8 × 32
Fault classification module $G_{P}$	Fully connected	Linear 2-1	8 × 32	8 × 8
		ReLU	/	8 × 8
		Linear2-2	3 × 8	8 × 3
		SoftMax	/	8 × 3
Domain discrimination module $G_{D}$	Fully connected	Linear 3-1	16 × 32	8 × 16
		LayerNorm	/	8 × 16
		ReLU	/	8 × 16
	Domain discrepancy measurement	Multi-Kernel	/	1

**Table 7:** Training configurations for cross-domain fault diagnosis tasks.

Configuration	Pre-Training	Domain Adaptation
	Value	
Optimizer	Adam	Adam
Batch size	8	8
Number of epochs	50	50
Learning rate of *G_G_*	0.005	5 × 10^−4^
Learning rate of other modules	0.005	3 × 10^−3^
Learning-rate decay	/	1 × 10^−4^ after epoch 40

**Table 8:** Mean fault identification accuracy (%) of different methods under cross-domain tasks.

Task ID	Identification Accuracy (%)			
	Optimized CNN	TCNN	DANN	Proposed Method
$T_{A1}$	79.1	87.9	92.5	94.6
$T_{A2}$	76.8	93.8	94.8	95.5
$T_{A3}$	75.4	90.4	87.2	94.4
$T_{A4}$	77.2	94.0	89.5	97.6
$T_{A5}$	80.3	92.6	92.5	92.8
$T_{A6}$	83.0	89.2	91.8	96.1
$T_{B1}$	66.4	84.1	88.4	94.1
$T_{B2}$	63.1	85.6	82.7	89.4
$T_{B3}$	58.7	83.9	82.6	87.4
$T_{B4}$	51.6	89.1	87.7	89.6
$T_{B5}$	65.2	80.5	87.8	90.0
$T_{B6}$	59.8	84.6	83.4	93.2
Mean value	69.7	87.9	83.4	92.9

**Table 9:** Accuracy (%) of different split mechanisms under cross-domain tasks.

Task ID	Accuracy (%)		
	Random Split	Block-Wise Split	Difference
$T_{A1}$	94.6	93.2	−1.4
$T_{A2}$	95.5	93.0	−2.5
$T_{A3}$	94.4	92.6	−1.8
$T_{A4}$	97.6	96.7	−0.9
$T_{A5}$	92.8	90.5	−2.3
$T_{A6}$	96.1	94.5	−1.6
$T_{B1}$	94.1	91.1	−3.0
$T_{B2}$	89.4	87.5	−1.9
$T_{B3}$	87.4	83.9	−3.5
$T_{B4}$	89.6	87.0	−2.6
$T_{B5}$	90.0	88.8	−1.2
$T_{B6}$	93.2	90.4	−2.8
Mean value	92.9	90.8	−2.1

**Table 10:** Ablation results of different components of FE-MK-DANN on *T_A_*_4_, *T_B_*_3_, and *T_B_*_5_.

Method Variant	Identification Accuracy (%)			
	$T_{A4}$	$T_{B3}$	$T_{B5}$	Average
w/o Attention	95.2	85.3	88.6	89.7
Plain Conv. (w/o Residual)	95.6	85.8	89.0	90.1
Single-kernel MMD	94.8	83.8	87.9	88.8
Adversarial only	93.6	82.5	87.1	87.7
MK-MMD only	94.1	81.7	86.9	87.6
End-to-end training	95.8	84.8	88.7	89.8
Full FE-MK-DANN (two-stage)	97.6	87.4	90.0	91.7

## Data Availability

The raw data supporting the conclusions of this study will be made available by the corresponding author upon reasonable request.

## Appendix A

The time-domain statistical features listed in Table A1 are extracted from the vibration signals, where $x_{i}$(i = 1,2,…,*N*) denotes the *i*-th discrete sample of the signal, and *N* is the total number of discrete samples.

**Table A1:** Time-domain statistical features of ball screw vibration signals.

Time-Domain Feature	Calculation Formula	Time-Domain Feature	Calculation Formula
Maximum value	$T_{1}=\max\left(x\right)$	Root mean square	$T_{10}=\sqrt{{\sum_{i=1}^{N}\left|x_{i}\right|}^{2}/N}$
Minimum value	$T_{2}=\min\left(x\right)$	Waveform factor	$T_{11}=T_{10}/T_{4}$
Mean	$T_{3}=\sum_{i=1}^{N}x_{i}/N$	Crest factor	$T_{12}=T_{5}/T_{10}$
Mean absolute value	$T_{4}=\sum_{i=1}^{N}\left|x_{i}\right|/N$	Impulse factor	$T_{13}=T_{5}/T_{4}$
Peak value	$T_{5}=\max\left(\left|x\right|\right)$	Clearance factor	$T_{14}=T_{5}/T_{14}$
Variance	$T_{6}=\sum_{i=1}^{N}{\left(x_{i}-T_{3}\right)}^{2}/N$	SRM waveform factor	$T_{15}=T_{14}/T_{4}$
Standard deviation	$T_{7}=\sqrt{\sum_{i=1}^{N}{\left(x_{i}-T_{3}\right)}^{2}/N}$	Fifth central moment	$T_{16}=T_{8}^{5/4}$
Kurtosis	$T_{8}=\sum_{i=1}^{N}{\left(x_{i}-T_{3}\right)}^{4}/N{\left(\frac{1}{N}\sum_{i=1}^{N}{\left(x_{i}-T_{3}\right)}^{2}\right)}^{2}$	Clearance	$T_{9}=\frac{1}{N}\sum_{i=1}^{N}{\left(x_{i}-T_{3}\right)}^{3}/{\left(\sqrt{\frac{1}{N}\sum_{i=1}^{N}{\left(x_{i}-T_{3}\right)}^{2}}\right)}^{3}$

The frequency-domain statistical features listed in Table A2 are extracted from the vibration signals, where $s\left(j\right)$ (j = 1,2,…,*M*) denotes the spectrum obtained by Fourier transforming the time-domain signal, and *M* is the number of discrete frequency points.

**Table A2:** Frequency-domain statistical features of ball screw vibration signals.

Frequency-Domain Feature	Calculation Formula	Frequency-Domain Feature	Calculation Formula
Mean	$F_{1}=\sum_{j=1}^{M}s\left(j\right)/M$	E-index	$F_{9}=\sum_{j=1}^{M}f_{j}^{2}s\left(j\right)/\sqrt{\sum_{j=1}^{M}s\left(j\right)\sum_{j=1}^{M}f_{j}^{4}s\left(j\right)}$
Variance	$F_{2}=\sum_{j=1}^{M}{\left(s\left(j\right)-F_{1}\right)}^{2}/\left(M-1\right)$	G-index	$F_{10}=F_{6}/F_{5}$
Third central moment	$F_{3}=\sum_{j=1}^{M}{\left(s\left(j\right)-F_{1}\right)}^{3}/M{\left(\sqrt{F_{2}}\right)}^{2}$	Third central moment	$F_{11}=\sum_{j=1}^{M}{\left(f_{j}-F_{5}\right)}^{3}s\left(j\right)/\left(M\;F_{6}^{3}\right)$
Fourth central moment	$F_{4}=\sum_{j=1}^{M}{\left(s\left(j\right)-F_{1}\right)}^{4}/M{\left(\sqrt{F_{2}}\right)}^{2}$	Fourth central moment	$F_{12}=\sum_{j=1}^{M}{\left(f_{j}-F_{5}\right)}^{4}s\left(j\right)/\left(M\;F_{6}^{4}\right)$
Overall mean	$F_{5}=\sum_{j=1}^{M}f_{j}s\left(j\right)/\sum_{j=1}^{M}s\left(j\right)$	J-index	$F_{13}=\left(F_{7}+F_{8}\right)/F_{1}$
Standard deviation	$F_{6}=\sqrt{\sum_{j=1}^{M}{\left(f_{j}-F_{5}\right)}^{2}s\left(j\right)/M}$	First-order spectral kurtosis	$F_{14}=M\sum_{j=1}^{M}s{\left(j\right)}^{4}/{\left(\sum_{j=1}^{M}s{\left(j\right)}^{2}\right)}^{2}$
C-index	$F_{7}=\sqrt{\sum_{j=1}^{M}f_{j}^{2}s\left(j\right)/\sum_{j=1}^{M}s\left(j\right)}$	Frequency-band amplitude	$F_{15}=\{s\left(p-a\right)\text{\textasciitilde{}}s\left(p+a\right)\}$
D-index	$F_{8}=\sqrt{\sum_{j=1}^{M}f_{j}^{4}s\left(j\right)/\sum_{j=1}^{M}f_{j}^{2}s\left(j\right)}$		
